# Exploring the transportome of the biosurfactant producing yeast *Starmerella bombicola*

**DOI:** 10.1186/s12864-021-08177-x

**Published:** 2022-01-09

**Authors:** Silke Claus, Sylwia Jezierska, Liam D. H. Elbourne, Inge Van Bogaert

**Affiliations:** 1 Centre for Synthetic Biology, Department of Biotechnology, Ghent University, Coupure Links 653, 9000 Ghent, Belgium; 2 Department of Molecular Sciences, Macquarie University, Macquarie Park, NSW 2109 Australia

**Keywords:** *Starmerella bombicola*, Transportome, Sophorolipid, Yeast, ABC transporters

## Abstract

*Starmerella bombicola* is a non-conventional yeast mainly known for its capacity to produce high amounts of the glycolipids ‘sophorolipids’. Although its product has been used as biological detergent for a couple of decades, the genetics of *S. bombicola* are still largely unknown. Computational analysis of the yeast’s genome enabled us to identify 254 putative transporter genes that make up the entire transportome. For each of them, a potential substrate was predicted using homology analysis, subcellular localization prediction and RNA sequencing in different stages of growth. One transporter family is of exceptional importance to this yeast: the ATP Binding Cassette (ABC) transporter Superfamily, because it harbors the main driver behind the highly efficient sophorolipid export. Furthermore, members of this superfamily translocate a variety of compounds ranging from antibiotics to hydrophobic molecules. We conducted an analysis of this family by creating deletion mutants to understand their role in the export of hydrophobic compounds, antibiotics and sophorolipids. Doing this, we could experimentally confirm the transporters participating in the efflux of medium chain fatty alcohols, particularly decanol and undecanol, and identify a second sophorolipid transporter that is located outside the sophorolipid biosynthetic gene cluster.

**Supplementary Information:**

The online version contains supplementary material available at 10.1186/s12864-021-08177-x.

## Background

Phospholipid-based cell membranes facilitate the possibility of cellular life, but at the same time create the need for the cell to access to the exterior environment; this need is provided for by membrane transporter proteins. Their crucial roles range from nutrient uptake to export of undesired product, from cell volume control to extracellular nutrient sensing and much more [1]. The set of genes encoding proteins contributing to this purpose is called the ‘transportome’. The metabolic cost for keeping the transportome operational cannot be underestimated; up to 60% of the total ATP requirement of the cell is required for activity of the transportome [2]. Transport reactions account for roughly one third of the reactions in the consensus yeast metabolic network reconstruction [3]. Yet, despite their apparent importance, membrane transporters remain widely neglected [4, 5]. Analogous to the Enzyme Commission (EC) system, the Transport Classification Database (http://www.tcdb.org) aims to organize membrane transport proteins based on both functional and phylogenetic information [6]. Different types of transport proteins are separated into classes, subclasses, superfamilies, families and subfamilies. Three main classes of transporters can be distinguished. Channels or pores (TC.1) create an aqueous pathway for polar compounds of a specific size and charge to pass through the membrane. The transport rates are high, up to 10^8^ molecules per second, and are driven by the electrochemical gradient of the molecule, in an energy-independent process. Carriers (TC.2) bind their substrate and catalyze its crossing after conformational change without the need for utilization of a primary source of energy. This can be for a single substrate, termed ‘uniporter’, or two or more substrate species in the opposite direction, termed ‘antiporter’ or two or more substrate species in the same direction, termed ‘symporter’. Antiporters and symporters are ‘secondary active’ systems; the energy stored in the electrochemical gradient of one of the two substrate species drives the other substrate against its electrochemical gradient. The largest and best-known superfamily within this class is the Major Facilitator Superfamily (MFS) of transporters [7]. They are single-polypeptide secondary carriers involved in symport, antiport or uniport of a vast array of substrates, across all kingdoms of life. The pumps (TC.3) are ‘primary active’ transporters and use the hydrolysis of ATP to drive active transport of the substrate against its gradient. The largest and best-known superfamily of this class are the ATP-Binding Cassette (ABC) transporters. Together with the MFS superfamily, this ABC superfamily accounts for nearly half of the known solute transporters. The TC.9 designation is used for transporters that have an incompletely characterized transport system. Mechanisms of bulk transport via endocytosis/exocytosis and intracellular trafficking are however not included in the TCDB and are therefore not discussed in this manuscript.

**Fig. 1:**
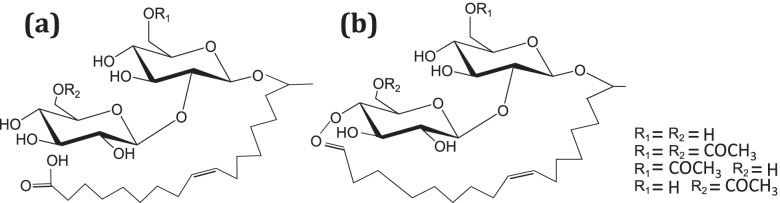
Generic chemical structure of (**a**) acidic sophorolipids and (**b**) lactonic sophorolipids

The non-conventional yeast *Starmerella bombicola* is mostly known for its high capacity (> 300 g/L) for producing sophorolipids (SL; Fig. 1) as secondary metabolites [8]. Despite the fact that it was discovered five decades ago, and is used in an industrial setup for the production of its sophorolipid biosurfactants, relative little is known about its genetics. Four genome assemblies of this haploid yeast are publicly available in the NCBI database (GCA_001599315.1, GCA_004124885.1, GCA_000950655.1, GCA_003033785.1) yet annotation is lacking. Few genes are characterized resulting in 343 nucleotide sequence records (NCBI database consulted at 12-03- 2021). Roughly 10% thereof is characterized in depth because of their role in sophorolipid biosynthesis and degradation or in the lipid metabolism [9]. Most importantly, the 13-kb long SL biosynthetic cluster with its central SL transporter ‘Mdr’ was found at the end of chromosome 2, and is well described [10]. Its expression pattern in the stationary phase was attributed to the subtelomeric position of the cluster and associated regulatory elements [11]. Recently, our group identified two mitochondrial citrate carriers and the ATP citrate lyase, and demonstrated their importance in sophorolipid production [12]. Furthermore, two fatty acid importers located in the peroxisome membrane have been described [13]. Besides being a notoriously good biosurfactant producer, *S. bombicola* has other exceptional features that involve compounds crossing the lipid bilayers; it can easily take up hydrophobic compounds such as fatty acids, alkanes, fatty alcohols and hydroxylated fatty acids [14, 15]. Furthermore, it is exceptionally resistant to a wide range of antibiotics, even though it is not pathogenic [16]. This manuscript presents a complete inventory of *S. bombicola*’s transporters gathered through sequence-based analysis, complemented by selected functional studies in search for key transport processes.

## Results

### *Identification of the* S. bombicola *transportome*

**Fig. 2:**
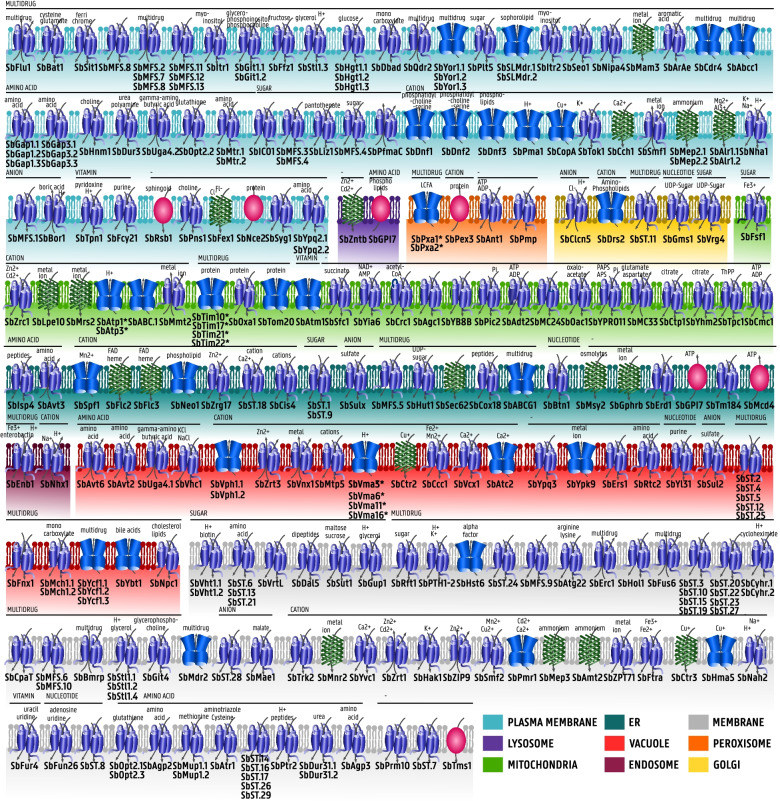
Overview of the predicted transporters in S. bombicola with their substrates predicted according to homology. Pumps are depicted in blue, channels in green and carriers in purple. Transporters of unknown mechanism are depicted as green oval. The color of the membrane structure represents the subcellular localization of the transporter as predicted by the DeepLoc online tool and curated by the experimental evidence of the closest homologs. Transporters are grouped according to the substrate specificity found by the TransAAP algorithm: amino acids, multidrug, cations,anions,vitamin, nucleotides and sugar. More detailed information on the substrate groups can be found in the Supplementary Table S2

**Fig. 3:**
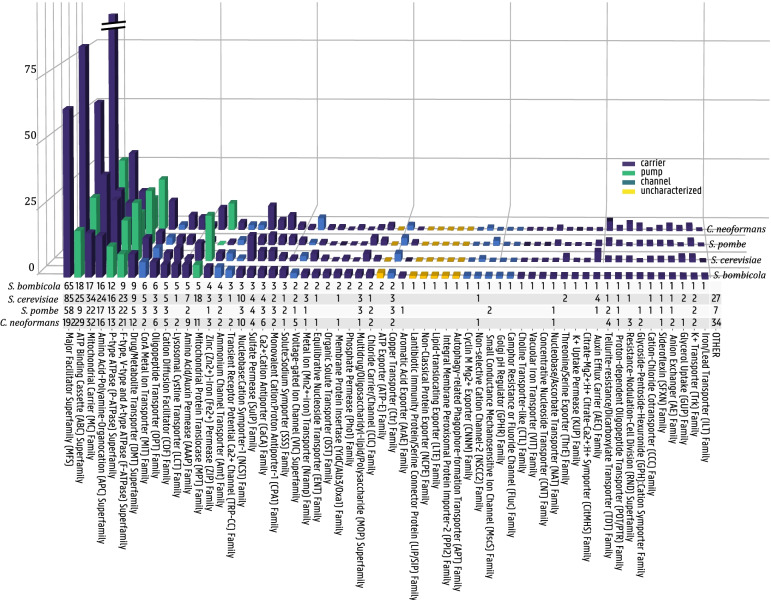
Transporter (super) family distribution of predicted transporters of S. bombicola in comparison with *S. cerevisiae*, *S. pombe* and *C. neoformans* according to the TCDB classification system

The 4626 predicted protein sequences in the *S. bombicola* genome were scanned with the transporter identification pipeline [17]. 254 genes were identified as putative transporter genes and subsequently subjected to sequence-based functional grouping: 180 genes were found to be of the type ‘channels’ (TC.1), 24 of the type ‘carrier’ (TC.2) and 44 of type ‘pump’ (TC.3). 7 genes belong to the group of incompletely characterized transport systems. For a complete list of the putative transporter gene designation to Saier’s TC system and their predicted function, the reader is referred to the additional file. All transporters, their class, their substrate group, potential substrate and subcellular localization are summarized in Fig. 2. In Fig. 3, the distribution of predicted transportome members among different (super)families is given. By means of comparison, the number of family members in the three yeasts present in TransportDB, *Saccharomyces cerevisiae, Schizosaccharomyces pombe* and *Cryptococcus neoformans,* is given as well. According to this analysis, the *S. bombicola* transportome accounts for 5.49% of its genome (*S. cerevisiae* 5.68%) with a transporter gene density of 26.75 transporter genes per Mb (*S. cerevisiae*: 28.05). These proportions accord with what is found for the fungal branch of transportome analyses throughout all living cells [2]. The largest superfamily within the *S. bombicola* transportome is the MFS superfamily (TC 2.A.1) with 65 members, clustered into 15 families, most notably the Sugar Porter Family (TC 2.A.1.1) with 15 members and the Drug:H+ Antiporter-1 (DHA1) Family (TC 2.A.1.2) with 11 members.

### *Mapping the expression profile of the* S. bombicola *transportome*

**Fig. 4:**
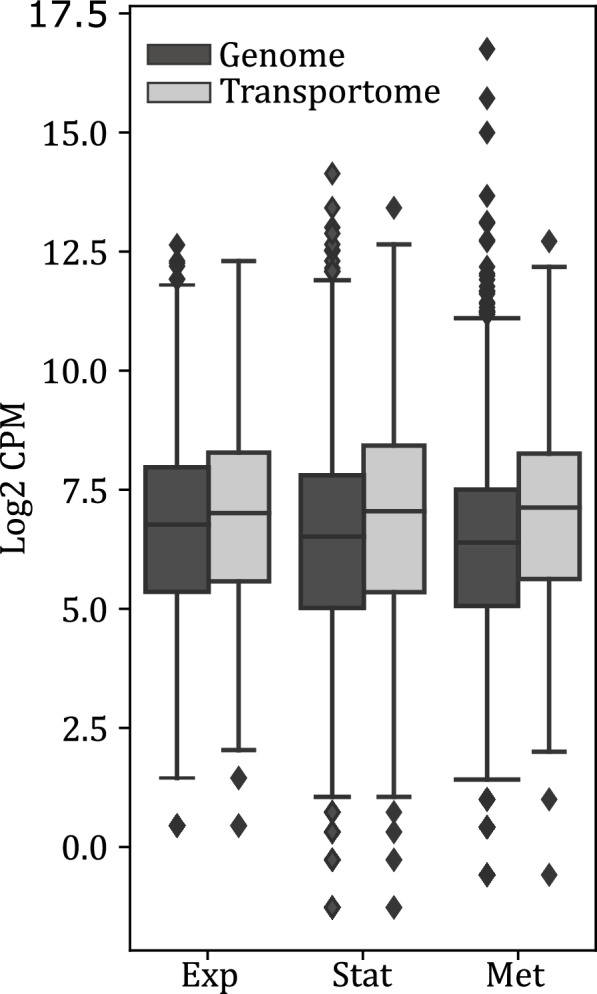
RNA Seq Count Data distribution after TMM normalization of the entire genome compared to the data distribution for the transportome. ‘Exp’: data sampled in the exponential phase, ‘Stat’: data samples in the stationary phase and ‘Met’: data sampled very late in the growth stage where the assembled SLs are the sole carbon source left. The horizontal line within the box represents the median, the lower and upper boundary of the box represent the first and third quantile respectively. Whiskers above and below the box extend to the most extreme data point which is no more than 1.5 times the interquantile range. Outliers beyond this range are represented by diamonds

It is unlikely that the entire transportome is present in the yeast cell’s membrane at any given time. Rather, subsets of the transportome are expressed according to their function in the yeast’s growth phase or environmental conditions. Whole-cell RNA sequencing was performed in three different growth stages in order to evaluate the expression time frame of the transportome: (1) in the exponential growth phase, (2) in early stationary phase when SLs are produced as a kind of secondary metabolite and (3) very late in the fermentation process, when all carbon sources are depleted except for the extracellularly produced SLs, which are being metabolized at that stage. From Fig. 4, it is clear that in all three growth stages, the RNA level per gene in the transporter subset is higher than the average RNA level in the complete *S. bombicola* gene set. SbStl1.1 and SbVht.1 appear to be inactive in the tested growth stages since expression was not detected.

**Fig. 5:**
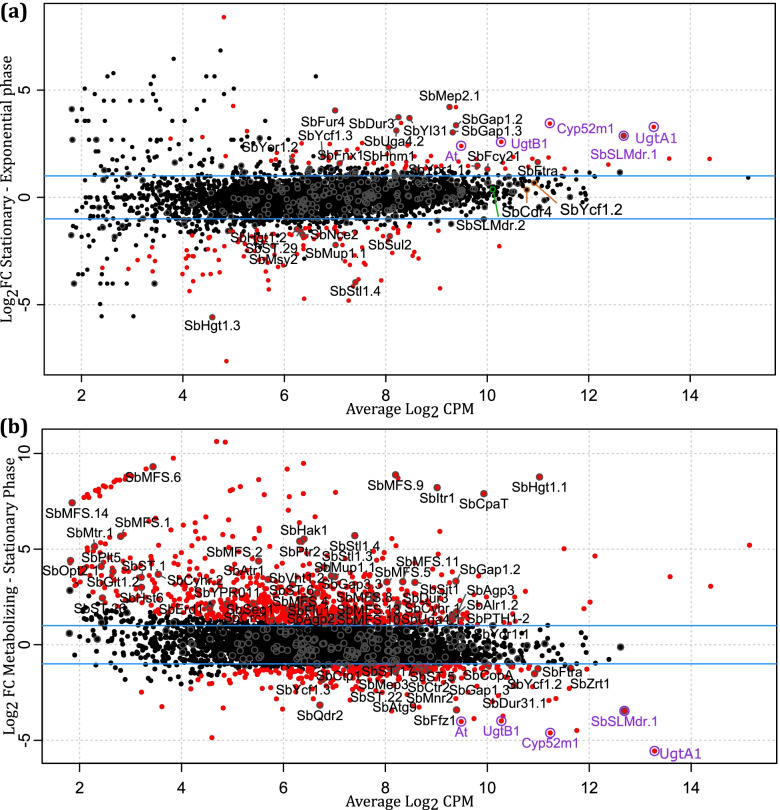
Smear plot showing the log2 fold change (FC) versus the average log2 count per million (CPM) of the change in gene expression. **a** Genes with positive Log2 FC are upregulated in the stationary phase compared to the exponential phase, while genes with negative FC are downregulated. **b** Genes with positive FC are upregulated under metabolizing conditions compared to the stationary phase, while genes with negative FC are downregulated. The horizontal blue lines delineate log2 FC ≥1, indicating genes with two fold differences in expression. Each dot represents one gene, transporters present in the predicted transportome are circled in grey. Genes from the SL biosynthetic gene cluster are indicated in purple. Differentially expressed genes (*FDR < 0.05) *are indicated in red

The datasets were analyzed to find differentially expressed genes in the three growth stages (Fig. 5). The large majority of transporters has a Log2 Fold Change (FC) of around 0 when comparing the exponential growth phase with the stationary growth phase, indicating that expression levels do not change over time (Fig. 5a). Yet, 23 transporters display a significant change. Transporters associated with growth are indeed more represented in the exponential phase. Most obviously the sugar uptake carriers SbHgt1.3, SbStl.4, SbHgt1.2, the amino acid uptake carriers SbMup1.1, SbST.29 and sulfate uptake carrier SbSul2. Towards the stationary phase, central cellular processes are slowed down, and other transporters play a bigger role. The most striking differentially expressed one is the SbSLMdr.1. Together with the other SL biosynthetic genes, this transporter is highly upregulated in the stationary phase. Furthermore, transporters implicated in nitrogen metabolism are upregulated under SL producing conditions. SbMep2.1 channeling ammonium, SbDur3 carrying urea and polyamines, SbFur4 for uracil, SbYl31 and SbFcy21 purines. Also, the amino acid transporter orthologues SbGap1.2 and SbGap1.3 are upregulated. SbFtra is highly expressed, suggesting an active iron uptake in this later growth phase. It is also in this stationary phase that most multidrug transporters (SbYor1.1, SbYor1.2, SbFnx1, SbYcf1.3) are being expressed. In Fig. 5b, gene expression very late in the fermentation stage (i.e. ‘metabolizing’ conditions) is compared with the gene expression in the early stationary phase. In this metabolizing stage, all carbon sources are depleted, and the produced SLs are hydrolyzed and metabolized for the cell’s survival. There is a large shift in the expression profile: 80 of the 254 transporter genes (31.50%) are significantly differentially expressed, 1029 of the 4626 genes (22.24%) on genome level. The most remarkable differences are found in the Major Facilitator Superfamily, where the presumed sugar transporters SbHgt1.1 and SbItr1 stand out. Many other members of this family with unknown substrate preference are also upregulated (SbStl1.4, SbCpaT and SbMFS.1-14, except SbMFS.3, SbMFS.7, SbMFS.12). Important members of the ABC transporter family on the other hand (most prominently SbSLMdr.1, but also SbYcf1.2-3), are downregulated. Also the nitrogen transporters highly upregulated in the stationary phase are now significantly downregulated.

### Highlighting the ABC transporter family

*S. bombicola* is not known to cause any disease, and consequently is not exposed to antibiotics other than the ones in its natural environment. Yet, it shows a remarkably high resistance to a variety of antibiotics and antifungals. The multidrug resistance phenotype is driven by a complex interactive network of transcriptional regulators and ABC efflux pumps. In the model yeast *S. cerevisiae*, the main detoxification transporters are Pdr5, Snq2 and Yor1, all members of the ABC Superfamily [18]. The physiological functions performed by yeast ABC transporters are diverse, and include mating factor secretion, regulation of mitochondrial function, vacuolar detoxification and pleiotropic drug resistance [19]. Furthermore, they are involved in the transport of hydrophobic molecules [20]. Which means that these transporters are also likely candidates to explain why *S. bombicola* is exceptionally good at export of fatty acids and derivatives thereof [14, 21].

In total, 18 members of the ABC Superfamily are retrieved in the *S. bombicola* transportome, including the described SL transporter SbSLMdr.1 and two peroxisomal fatty acid importers SbPxa1 and SbPxa2 [10, 13]. 12 out of the 18 are annotated as ‘full size’ transporters (2 Nucleotide Binding Domains (NBD) and at least 2 Transmembrane Domains (TMD)) and 5 as ‘half size’ transporters (1 NDB and 1 TMD). One transporter, SbABC.1, is predicted to be a close homolog of *S. cerevisiae* YDR061W, a protein which remarkably lacks membrane-spanning regions, yet is an uncharacterized family member of ABC transporters. All putative ABC transporters are listed in Table 1.

**Table 1:** Predicted *S. bombicola* ABC transporters. Closest homologs are represented by their UniProtKB accession number, followed by its systematic name between brackets when *S. cerevisiae* is the yeast of origin

ID	Size (AA)	Topology			TCDB no.	Subcellular location	Closest homolog
		NBD	TMD	TMH			
SbSLMdr.1	1299	2	2	12	3.A.1.201	Plasma membrane	H6TB12
SbSLMdr.2	1331	2	2	12	3.A.1.201	Plasma membrane	H6TB12
SbHst6	1261	2	2	12	3.A.1.206	Membrane	P53706
SbYbt1	1626	2	3	17	3.A.1.208.12	Vacuole	P32386 (YLL048C)
SbYcf1.1	1497	2	3	17	3.A.1.208.11	Vacuole	P39109 (YDR135C)
**SbYcf1.2**	1480	2	3	17	3.A.1.208.11	Vacuole	P39109 (YDR135C)
**SbYor1.1**	1445	2	2	12	3.A.1.208.3	Plasma membrane	P53049 (YGR281W)
**SbYor1.2**	1358	2	2	12	3.A.1.208.3	Plasma membrane	P53049 (YGR281W)
SbAbcc1	1231	2	2	10	3.A.1.208	Plasma membrane	O35379
SbYcf1.3	1450	2	3	16	3.A.1.208.11	Vacuole	P39109 (YDR135C)
SbYor1.3	1361	2	2	12	3.A.1.208.3	Cell membrane	P53049 (YGR281W)
**SbCdr4**	1543	2	2	13	3.A.1.205	Plasma membrane	O74676
SbMdr2	718	1	1	6	3.A.1.201	Membrane	Q4WPP6
SbAtm1	561	1	1	4	3.A.1.210	Mitochondrial membrane	Q6CX96
SbABC.1	469	0	0	0	3.A.1	Mitochondrial membrane	Q12298 (YDR061W)
SbABCG1	1322	1	1	7	3.A.1.204	Endoplasmic reticulum	D4AYW0
SbPxa2	698	1	1	5	3.A.1.203.6	Peroxisome membrane	P34230 (YKL188C)
SbPxa1	746	1	1	5	3.A.1.203.6	Peroxisome membrane	P41909 (YPL147W)

**Fig. 6:**
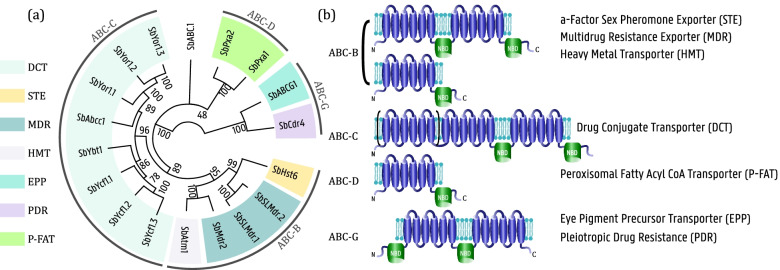
**a** The maximum-likelihood phylogenetic tree using 1000 replicates of the ABC Superfamily members in *S. bombicola* transportome*,* after alignment using Praline (https://www.ibi.vu.nl/programs/pralinewww/)*.* Gene names are given at the terminal nodes. Subfamily Classification according to the Human Genome Organization (ABC-A to ABC-H) as well as the TCDB family designation is indicated (see legend) **b** Predicted topology according to their HUGO subfamily grouping and confirmed by sequence analysis by the Scan Prosite online tool (http://prosite.expasy.org). TMDs consists of six transmembrane helices, represented in blue. NBDs with the Walker A and B motifs and the ABC signature motif are represented in green

The 18 ABC transporters segregate into seven subfamilies, according to the phylogenetic tree pictured in Fig. 6a and sequence comparison with their closest homologs. An alternative classification according to the Human Genome Organization (HUGO), where all eukaryotic ABC proteins are subdivided in eight subfamilies ABC-A to H, is also given. Figure 6b shows the predicted topology for every subfamily member. The largest subfamily within the ABC transporters, with 8 members, is the Drug Conjugate Transporter family (DCT) or more commonly known as the multidrug resistance protein (MRP) family. This is in contrast to other yeasts such as *S. cerevisiae* or *C. albicans,* where the PDR subfamily is largest [22]. The entire fungal ABC-C subfamily of ABC transporters is very poorly characterized except for some of *S. cerevisiae* transporters, most notably *Yor1* and *Ycf1,* both responsible for drug/drug conjugate resistance by export into the extracellular medium or vacuolar sequestration respectively. In the ABC-B group, one can find an orthologue to the first ABC transporter ever to be characterized, SbHst6, orthologous to the *S. cerevisiae STE6* and responsible for the export of the farnesylated peptide pheromone a-factor [23]. This subfamily contains both full-size and half-size transporters, and have a wide variety of functions [24]. ABC-D transporters are half-size transporters that dimerize at the peroxisomal membrane, where they allow fatty acids to enter the peroxisome in the form of acyl-CoA [25]. *S. bombicola* has, like *S. cerevisiae*, two members of this family [13]. The ABC-G subfamily can be distinguished from the others on regard of their ‘reverse orientation’: the signature NBD precedes the TMD domains instead of the more common ‘forward orientation’ where a TMD is most N-terminal. In the *S. cerevisiae* genome, 10 ABC-G members can be found. All of them are intensively studied because of their involvement in the pleiotropic drug resistance (PDR) phenotype. Curiously, this subfamily is rather underrepresented in *S. bombicola,* with only SbCdr4 and SbABCG1 predicted to export a range of drugs.

Four transporters were selected to proceed with functional studies: SbYcf1.2, SbYor1.1, SbYor1.2 and SbCdr4. All four are full size transporters with a clearly predicted topology and localization at the plasma membrane, which flags them as candidates for participation in drug tolerance and hydrophobic compound export [26, 27]. Besides homology to *Candida albicans CDR4*, SbCdr4 shows high similarity to the *Y. lipolytica* ABC1 alkane transporter [28]. SbYcf1.2 is also predicted to be situated at the vacuole membrane, indicating an alternative manner of detoxification, by sequestration in the vacuole. Founded on their similarity to *S. cerevisiae YOR1,* SbYor1.1 and SbYor1.2 are predicted to export a range of drugs as well as phospholipids [29, 30]. The differential expression analysis in Fig. 4 showed that both are significantly upregulated in the stationary phase. SbYcf1.2 and SbCdr4 are highly expressed throughout the yeast’s entire life cycle. Only three ABC proteins are essential for survival of *S. cerevisiae:* ScYef3p, ScArb1p and ScRli1p, of which none are involved in transport [31]. The mitochondrial ABC transporter ScAtm1 is essential for respiratory function and hence for survival in aerobic conditions [32]. Creating ABC transporter knockout mutants of the non-essential ABC transporters proved to be a valuable method to understand their function in *S. cerevisiae* [33]. Hence, single gene deletions were made for *S. bombicola* as well. Firstly, the viability of the knockout mutants was checked and appeared to be affected compared to wild-type *S. bombicola*, but not significantly different from each other (see Table S5)*.*

**Fig. 7:**
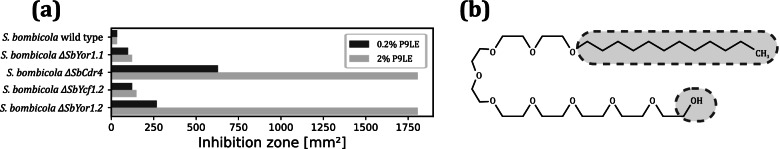
**a** The inhibitory effect of polyoxyethylene 9 lauryl ether (P9Le) as represented by the area of clearing zone [mm^2^] **b** Graphical representation of the chemical structure of P9Le. Dashed lines indicate the potential transport recognition sites

Secondly, the knockout mutants were subjected to a disc diffusion assay, applying a list of structurally unrelated compounds in various concentrations to assess their potential role in the export of those compounds (Table S6). From these results, no clear difference between wild-type and the deletion mutants could be observed in the transport of several flavonoids, aniline, rhodamine B and 2-nitrophenol. The antimicrobials G418, hygromycin, chloramphenicol, nystatin and phleomycin also did not differentiate the different strains. Nor did the detergents Triton X-100 and Triton X-144 result in inhibitory effect. Lauroylsarcosine was equally toxic to all strains. Interestingly, the mild non-ionic detergent polyoxyethylene-9-lauryl ether (P9Le) had a strong inhibitory effect on *S. bombicola ΔSbCdr4* and *S. bombicola ΔSbYor1.2,* associated with increasing concentration, whilst only a minor toxic affect was observed in *S. bombicola ΔSbYor1.1* and *S. bombicola ΔSbYcf1.2.* Wild-type growth was not affected at all (Fig. 7a). P9Le consists of a long carboxyl group linked to a long ethylene oxide chain (Fig. 7b).

**Fig. 8:**
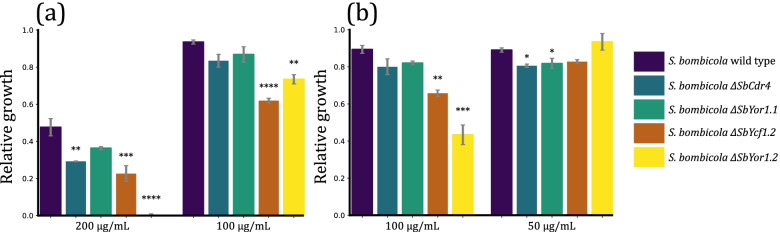
Relative growth of *S. bombicola* ABC transporter knockout mutants in SD medium supplemented with **a** decanol and **b** undecanol. The results are shown as mean ± standard error of the mean. Statistically significant differences compared to wild-type relative growth are indicated with asterisks. * *p* < 0.05, ** *p* < 0.01, *** *p* < 0.001, **** *p* < 0.0001

These results indicate a hydrophobic substrate preference for both SbCdr4 and SbYor1.2. Therefore, the toxicity of medium chain alkanes and fatty alcohols were tested in a next screening round in a disc diffusion assay (Fig. S2). A strong inhibitory effect of medium chain fatty alcohols was observed for *S. bombicola ΔSbCdr4* and *S. bombicola ΔSbYor1.2*. The disc diffusion assay was followed by toxicity tests in liquid culture based on the outcome of the fast screening technique (Fig. 8). The results are expressed as relative growth, calculated as OD_600_ measured after 48 h corrected by OD_600_ without the addition of the tested compound. It can be seen that all strains show decreased growth with the addition of 200 μg/mL decanol, most significantly in *S. bombicola ΔSbYor1.2*, where growth was completely abolished. *S. bombicola ΔSbCdr4* and *S. bombicola ΔSbYcf1.1* showed similar growth inhibition. Also for undecanol, *S. bombicola ΔSbYor1.2* seems the most sensitive among tested ABC transporter knockouts, followed by *ΔSbYcf1.1.* To conclude, these results show that *SbCdr4, SbYcf1.1* and *SbYor1.2* all contribute to the efflux of medium chain fatty alcohols decanol and undecanol with high substrate specificity.

### SLMdr.2: the second SL transporter

**Fig. 9:**
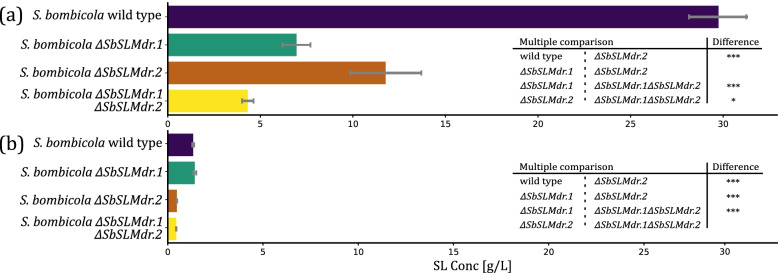
Multiple comparison of mean SL production on shake flask scale (**a**) with the addition of 37.5 g/L rapeseed oil and (**b**) without the addition of rapeseed oil. Statistically significant differences between wild type and SL transporter deletion mutants are indicated with asterisks, as calculated using one-way analysis of variance (ANOVA) with Tukey’s multiple comparisons test. * *p* < 0.05, ** *p* < 0.01, *** *p* < 0.001, **** *p* < 0.0001

Previous research demonstrated that the SbSLMdr.1, present in the SL biosynthetic gene cluster, is indeed largely responsible for export of SLs [10]. Being a member of the MDR subfamily of ABC transporters, it shows high similarity to known multidrug pumps, yet it does not appear to contribute to the high resistance phenotype with phleomycin, G418 and zeocin tested [10]. Quantification of SL production after deleting this transporter showed a large reduction in SL yield, never reaching more than 10% of wild-type strains. The transportome analysis uncovered a similar transporter in the *S. bombicola* genome, named SbSLMdr.2. This gene translates to 1331 amino acids (AA) which corresponds to a molecular weight of 146 kDa without considering post-translational modifications. In comparison; SbSLMdr.1 is 1299 AA in length, and 142 kDa. Alignment of both AA sequences results in an alignment length of 1345, with 59.18% AA identity and 72.57% AA similarity. In order to confirm the potential complementary role that SbSLMdr.2 might have to SL export, *S. bombicola* knockout mutants were created: *S. bombicola ΔSbSLMdr.1, S. bombicola ΔSbSLMdr.2* and *S. bombicola ΔSbSLMdr.1ΔSbSLMdr.2.* SL production was evaluated by performing a growth experiment with and without the addition of 37.5 g/L rapeseed oil to the culture broth. Rapeseed oil is commonly added to SL production medium because its triglycerides contain mostly oleic acid, which in turn is the most prevalent hydrophobic backbone of wildtype SL mixtures. *S. bombicola* readily takes up these fatty acids and immediately incorporates them in the SL resulting in higher SL titers. The viability and relative growth among the different SL transporter deletion mutants was monitored by means of OD_600_ and pH measurements every 24 h and in both experiments, no differences could be observed between the strains (Fig. S3). The SL titers of the resulting end extracts are depicted in Fig. 9. When rapeseed oil was added to the culture medium, a highly significant drop in SL export could be observed between wild type and both single SL transporter knockouts. Furthermore, the double deletion mutant produced even less SLs, indicating functional redundancy of the two transporters. Different results are obtained without adding rapeseed oil. As expected, the overall yield is drastically lower. The extracellular medium of *S. bombicola ΔSbSLMdr.1* shake flasks contained the same amount of SLs compared to wild-type. *S. bombicola ΔSbSLMdr.2* on the other hand showed significantly less SL export, the same as the double deletion mutant.

## Discussion

### *Identification of the* S. bombicola *transportome*

The importance of transport processes i.e. the uptake of nutrients, flux of signaling molecules or the extrusion of toxic compounds is evident. In industrial biotechnology, tinkering with the main driver can lead to increased production rates [34], the usage of waste feedstocks [35] or alternate product export [36]. Fundamental knowledge about these transporters is essential, yet so far limited. The bioinformatic analysis presented in this article is the first step on the way to functionally characterize the *S. bombicola* transportome. The transporter percentage and its density is in line with what is expected for small eukaryotic transportomes, with 100-500 members [2]. Furthermore, the distribution among transporter families follows roughly the same subdivision as the three yeasts present in the TransportDB database. The largest (super) family by far is the MFS superfamily of secondary active channels. The primary active pumps, ABC transporters, are the second largest family, making up less than a third in size. This finding follows the evolutionary trend that was observed from prokaryotes to higher eukaryotes; low-energy-demanding transport classes occur more frequently in higher eukaryotes compared to prokaryotes because of an evolutionary selection pressure for lower energy [2].

Arguably the most challenging aspect of fully understanding the transportome in any given organism, is the designation of the substrates and their specificity fitting each transport system. Generally, this knowledge cannot be gathered in a high-throughput setup but has a high technical and resource-intensive demand. Preliminary computational analysis is therefore often valuable to start from. This in silico analysis identified 254 putative transporters, and linked them to their respective orthologous genes in better known yeasts. Curated database TCDB as well as RefSeq were searched for substrate information providing the first clue regarding their function (Table S2). To improve accuracy of substrate prediction, evolutionary information and the AAIndex as implemented in the web server ‘TrSSP: The Transporter Substrate Specificity Prediction Server’ helped to confirm the TransAAP division of the transporters in the substrate groups depicted in Fig. 2 [37]. Knowledge of a protein’s subcellular localization is an additional help in understanding its proper function.

### *Mapping the expression profile of the* S. bombicola *transportome*

RNA sequencing is a tool that is conceptually unbiased; mapping the reads to the genome sequence helped to identify ORFs and therefore correctly annotate genes, finetuning the results that were obtained from the automated annotation pipeline to 254 transporters. The expression of two transporters, SbStl1.1 and SbVht1.1 could not be detected, which could imply that specific growth conditions are needed for their expression or that they might be candidate pseudogenes. We believe the latter to be the most likely, because both have at least one gene duplicate. The expression of all other transporter genes not only confirmed their presence, but also indicate their potential biological relevance.

The transporters with highest expression in the exponential phase are mostly glucose transporters, transporters involved in adenine and methionine synthesis, sulfur metabolism and cell wall. Also in *S. cerevisiae,* these functionalities are clustered with a similar expression pattern [38]. Martinez et al. chose ScMup1 as reference transporter, which shows a similar expression pattern as SbMup1.1 in this analysis: it is highly expressed in the exponential phase only to go down in the stationary phase. Later in the growth curve, reaching the stationary phase and therefore SL production, transporters involved in nitrogen metabolism are more highly expressed. A similar observation was made in *Pseudomonas aphidis*, during the production of mannosylerythritol lipids, just as sophorolipids a type of glycolipid [39]. This can be explained by the high carbon to nitrogen ratio at that timeframe; transcriptional changes occur with the release of nitrogen catabolite repression [40]. Nitrogen depletion switches on the transcription of *S. bombicola*‘s nitrogen transporters (SbGap1.2, SbGap1.3, SbDur3, SbUga4.2, SbFur4, SbHnm1,SbMep2.1) similarly to what is seen in *Y. lipolytica* and *S. cerevisiae* [41]*.* The biggest effect of this high C/N ratio however, is observed on the SL biosynthetic genes, including the transporter SbSLMdr.1. This is supported by previous results and *S. bombicola*’s natural niche, in the midst of flowers, bees, and therefore high sugar content [10]. SLs are produced extracellular as excess carbon storage, unavailable for consumption by competitive microorganisms. Because of this, SL production is only observed in the stationary phase. Indeed, all genes present in the SL biosynthetic gene cluster are induced in the stationary phase [42]. The expression of the entire SL biosynthetic cluster is regulated simultaneously, at least partially by a subtelomeric effect [11]. How exactly the C/N ratio is steering the cluster regulation is still to be determined in detail.

After analysis of the differential expression between the metabolizing sample and the stationary phase sample in Fig. 4b, it is clear that the cells are in survival mode. Because all nutrients enter the cell via transport proteins, the cell heavily invests in the presence of transporters with a large variety in capacity and affinity to increase the chances of gating the scarce nutrients that are left in the media into the cell. The best example of this phenomenon was found in *S. cerevisiae* and its hexose transport family encoded by *HXT* genes [43]. This family of glucose transporters contains 20 members that are differentially expressed depending on the glucose concentration in the medium. In excess of glucose, low affinity transporters are prevalent, while they are downregulated to be replaced by high affinity transporters when glucose becomes limited. How this regulation occurs in cooperation with glucose sensors ScSnf3 and ScRgt2, on transcriptional level, but also on post-translational level, was reviewed by Bisson et al. [44]. Following this reasoning, it can be speculated that SbHgt1.1, SbItr1, SbCpaT and SbMFS.9 are high affinity transporters for *S. bombicola’*s preferred carbon source, glucose. SbHgt1.3 on the other hand, that is very highly expressed in the exponential phase, will more likely be a high capacity and low affinity glucose transporter. Besides glucose, *S. bombicola* is in search for other forms of carbon and/or energy, which is reflected by the upregulation of amino acid and peptide transporters SbPtr2, SbMtr.1, SbOpt2.1 and SbMup1.1.

The same nitrogen transporters, i.e. SbGap1.3, SbDur31.1, SbMep3, under influence of the nitrogen regulation are again repressed when the C/N ratio drops to growth-limiting conditions in the metabolizing phase. Yet, some orthologues of the same amino acid transporters are actually upregulated (SbGap1.2, SbGap3.3). Most likely, the cell turns to a second strategy besides the higher glucose affinity; it upregulates transporters that can scavenge amino acids as alternative carbon source [43]. More information on how amino acid transporters are degraded by endocytosis in response to nitrogen excess or starvation in *S. cerevisiae* and *Aspergillus* spp. can be found in the recent review of Barata-Antunes et al. [45]. The SLs that are stored extracellularly are now actively consumed for the cell’s survival, so the SL biosynthetic genes and its central transporter are largely silenced in response to this lower C/N ratio. Other members of the ABC transporter family are also significantly downregulated. This can be explained by the functional mechanism of ABC transporters in eukaryotes that are mainly – but not all, as was believed until recently [46] - exporters. MFS transporters belonging to the Drug:H+ Antiporter-1 Family and therefore mainly responsible for export, such as SbQdr2, are downregulated. The same holds true for SbFfz1; this transporter previously described by Goncalves et al. as a specific, low affinity and high capacity fructose transporter that allows a fructophilic metabolism [47]. SbCtp1, the mitochondrial citrate carrier that was linked to SL production is also downregulated in SL metabolizing conditions [12].

### Highlighting the ABC transporter family

Yeast ABC transporters are shown to translocate structurally and functionally unrelated molecules. These include fungicides, antibiotics, flavonoids, detergents etc. [18]. For this reason, this group of transporters is investigated first in the search for the multidrug resistance phenotype culprits. Even though this yeast is not pathogenic, overcoming the multi-resistance phenotype is still beneficial from a biotechnological point of view. In recent years, some progress has been made toward engineering this rather unconventional yeast, yet genetic modification of it is still in its infancy compared to the myriad of engineering tools that are available in model yeasts nowadays [9]. Only two dominant drug selective markers can be used and this in rather high concentrations: 500 μg/mL hygromycin or 600 μg/mL nourseothricin which can be counteracted by the introduction of the *hygR* gene or *nat* gene respectively [48]. Understanding and restricting this multidrug resistance can help the development of new selective tools that can be used for genetic modification. Members of the MDR family within the ABC Superfamily are also known to transport lipids, lipopolysaccharides and/or lipoproteins [49]. It is therefore not a surprise that both the previously identified SbSLMdr.1 and the newly discovered SbSLMdr.2 can be categorized in this family.

Four transporters were chosen to study more in detail: SbCdr4, SbYcf1.2, SbYor1.1 and SbYor1.2 From Table S5, it can be seen that the selected transporters are not essential for viability, but they do have an influence on the yeast’s fitness. The closest homolog to SbCdr4, *CDR4* from *C. albicans* is one of the seven full-sized members of the Pdr protein subfamily that is largely responsible for the notorious multidrug resistance of that yeast [27]. However, *CDR4* does not have a role in antifungal resistance, yet it does function as a phospholipid translocator [50]. The second highest homology is shared with *Yarrowia lipolytica* ABC1, the long chain (C14/C16) alkane importer. Three other highly similar *Y. lipolytica* ABC transporters - ABC2-ABC4 - were also suggested to be involved in alkane transport, both in- and outward [20]. SbYor1.2 in its turn shows the closest homology to *YOR1*-like transporters from *S. cerevisiae* that are known to export a broad range of substrates, including hydrophobic molecules [18]. Indeed, Cui et al. (1996) showed that *S. cerevisiae ΔYOR1* is especially sensitive to carboxyl group containing compounds such as propionic acid or reveromycin A [29].

Disc diffusion assays with a broad substrate range, including hydrophobic compounds, detergents and antimicrobials were performed with all four deletion mutants and wild-type as a reference (Table S6). Most compounds could not differentiate between the subjected strains. Especially in the case of antifungals, detoxification occurs by the combined action of several efflux pumps. Single transporter deletions therefore may not be sufficient to observe their involvement in the export for most of the compounds. Yet, some important difference were observed: *S. bombicola* wild type is still able to grow in the presence of 200 μg/mL decanol, and only moderately affected by 100 μg/mL undecanol, whereas the model yeast *S. cerevisiae* already stops growing at 50 μg/mL and 25 μg/mL respectively [51]. The liquid toxicity assay suggests that this four times higher tolerance to medium chain fatty alcohols is attributed to the activity of ABC transporters. This knowledge can be of use in an industrial biotechnology set-up: medium chain fatty alcohols are broadly used in various industries, yet are currently extracted from vegetable oils and animal fat. The transition to a more ecological, microbial production of fatty alcohols would be beneficial. To that end, genetically engineered *E. coli* was already developed in 2009 [52]. Better results were obtained more recently using the oleaginous yeast *Y. lipolytica,* that reached maximum undecanol production titers of 550 mg/L [53]. In these works, the aspect of export was not taken into account, leading to growth impairing accumulation of the fatty alcohols inside the cell [41]. Furthermore, costly extraction procedures prohibit upscaling to industrial levels. The chief reason for this neglect is the lack of functional knowledge of transporters. Hu et al. (2018) even turned to domain shuffling of human fatty alcohol transporters, to improve fatty alcohol secretion in *S. cerevisiae* [34]. The *S. bombicola* transporters described in this manuscript are the first experimentally confirmed transporters that participate in the efflux of medium chain fatty alcohols and could therefore be used when aiming at higher production and efflux of these compounds.

### SLMdr.2: the second SL transporter

Secondary metabolites are non-essential for growth and reproduction, but are commonly produced to function as communication signals. Transport systems are often found within gene clusters encoding their biosynthetic genes, being responsible for the secretion of the secondary metabolite where they can interact with other (micro) organisms [54]. Furthermore, several ABC-transporters associated with secondary metabolism are highly conserved among fungi [24]. All fungal glycolipid biosurfactants produced by yeasts that are characterized so far are also organized in such clustered architecture with a transporter in its center, as was recently reviewed by our group [55]. Taken together, it is not surprising that SLMdr.1 is under heavy transcriptional regulations that coincide with the rest of the cluster genes. From the RNAseq results depicted in Fig. 5, it can be clearly seen that the SLMdr.1 transporter gene expression is highly upregulated in the stationary growth phase, governed by a high C/N ratio (1757.52 cpm exponential vs 10,943.07 cpm stationary). In metabolizing conditions, when the C/N ratio drops and SLs are being consumed rather than produced, the SLMdr.1 centrally located in the SL biosynthetic gene cluster, is again largely downregulated, yet its expression remains rather high (781.68 cpm). Nevertheless, secondary metabolite clusters are among the most mobile genetic elements, driven by processes such as gene loss, duplication and horizontal gene transfer [56]. This might explain the existence of a paralogous gene, SbSLMdr.2, that has the same function but is subject to different regulatory rules. SbSLMdr.2 is more steadily expressed in all three studied stages of growth, albeit slightly - but not significantly- higher in the metabolizing stage (852.00 cpm exponential, 941.01 cpm stationary and 1479.31 cpm metabolizing). These results clearly indicate a different regulatory system between these two highly similar transporters. Transcription of SLMdr.1 most likely is regulated as part of the SL biosynthetic gene cluster, a mechanism that is up to now only partially resolved [11]. A high C/N ratio is needed for induction of the cluster enzymes, and at least partially, the subtelemoric positioning of the cluster has an effect. SLMdr.2, located on another chromosome not surrounded by any other SL biosynthetic enzymes is no subject to this cluster regulation.

Analysis of SL formation with either *S. bombicola ΔSLMdr.1* or *S. bombicola ΔSLMdr.2* demonstrated a drop in product titer to approximately one third of what is achieved with wild type *S. bombicola*. The double knockout strain yielded even less (17%) compared to both single SL transporter knockouts, which means there is redundancy in their functionality. This can also be derived from the fact that the drop in SL export is equally large for both knockouts. Based on the UPLC chromatograms, no shift in composition of the SL mixture could be observed compared to the SL mixture produced by wild-type *S. bombicola*, which indicates that there is no marked substrate preference within the SL spectrum for either of the transporters. Even in the double deletion mutant, SL molecules could still be detected in the extracellular environment. Alternative secretion routes are probably still in play. Most likely they are less specific and consequently less efficient. A different outcome can be observed when no rapeseed oil is added to boost SL production. As expected, the overall yield is much lower (5-10%). Moreover, the SL production titers of both wild-type *S. bombicola* and *S. bombicola ΔSbSLMdr.1* is approximately the same. Only in *S. bombicola ΔSbSLMdr.2* and *S. bombicola ΔSbSLMdr.1ΔSbSLMdr.2,* a reduction in SL titer could be observed. This suggests that when no rapeseed oil is added to the fermentation broth, the SL export is predominantly governed by SbSLMdr.2, as opposed to the SbSLMdr.1, the transporter present in the biosynthetic gene cluster. Altogether, these results confirm the hypothesized role of SLMdr.2 as a SL exporter.

## Conclusions

The present study brings forward a complete inventory of transporter genes present in the genome of *S. bombicola*, based on in silico prediction and sequence similarities with characterized transporters from closely related yeasts or other organisms. With the RNA sequencing results in three different life cycle stages, some insight is given on the global expression profile, and compared to the expression profile of the transportome. The ABC transporter superfamily was highlighted by the creation of five deletion mutants. Four were tested for their capacity to export a large variety of compounds, including hydrophobic molecules. *S. bombicola ΔSbCdr4* and *S. bombicola ΔSbYor1.2* showed a strong inhibitory effect by the detergent P9LE as well as the medium chain fatty alcohols decanol and undecanol. *S. bombicola ΔSbYcf1.1* showed decreased relative growth compared to wildtype in the presence of decanol. So far, no microbial transporter with an active role in medium chain fatty alcohol export was described. The findings from this manuscript can thus be used for industrial biotechnology purposes, where a transition to microbial fatty alcohol production would be beneficial, compared to the extraction from vegetable oils and animal fat that is used nowadays.

Furthermore, aside from the already established SL exporter present in the biosynthetic gene cluster, a second SL exporter was found by in silico analysis: SbSLMdr.2. SL production assays with the corresponding deletion mutant confirmed that SbSLMdr.2 is indeed the main exporter of SLs when no rapeseed oil is added to the production medium, and has a secondary role in SL export when oil is added.

The unravelling of the *S. bombicola* transportome also provides a route for further exploitation of *S. bombicola* as non-conventional microbial cell factory, by its contribution to the primary insight into its cellular capabilities for utilizing nutrients or extruding non-natural compounds.

## Methods

### Identification of transporter proteins

Genome sequencing, assembly, ORF prediction and primary annotation was performed as described before [42]. All the predicted protein sequences were then analyzed with the membrane transporter analysis tool TransAAP, to informatically identify putative transporters [17, 57]. A local version of this was run which allows for a more permissive inclusion of transporters than the publicly available version, to maximize the chances of characterizing novel transporters, and additionally utilizes eukaryotic reference sequences and HMMs which are not employed in the online version due to computational restraints. The 254 putative transporter sequences were implemented in OmicsBox version 1.2.4 for functional annotation by performing BLASTp analysis against the UniProtKB/Swiss-Prot database (version 2019 11). Classification according to the Transporter Classification Database (http://www.tcdb.org/) was performed using provided BLASTp results. The TOPCONS web server (http://topcons.net/) was used for prediction of membrane spanning regions. Subcellular localization of the putative transporter genes was predicted by DeepLoc-1.0 and compared with the input from the BLASTp results and the associated experimental data [58]. Sequences were deposited in GenBank under bioproject MZ671253-MZ671487. The fungal transportomes other than *S. bombicola* were retrieved from the TransportDB database [17]. ABC transporters were classified as such with HMMSearch 3.1b2 (February 2015), part of HMMER (http://hmmer.org/), in search for the HMM model ‘ABC_tran’ (Pfam accession PF00005). Top hits with a domain score higher than 50 and e-value below 1e-15 were withheld as true members of the ABC Superfamily. The amino acid sequences were used for phylogenetic analysis after alignment using Praline (https://www.ibi.vu.nl/programs/pralinewww/). A tree was inferred using the maximum-likelihood method bootstrapped with 1000 replicates implemented in MEGA X version 10.0.5. Amino acid identity and similarity is calculated after alignment with Clustal Omega with the online ‘Ident and Sim’ tool (https://www.bioinformatics.org/sms2/ident_sim.html).

### Strains and culture conditions

The *S. bombicola* strains used in this research are listed in Table S3. *Escherichia coli* Top10 cells (Invitrogen, Carlsbad, U.S.A.) were used for the purpose of cloning and propagation of plasmids. All *S. bombicola* strains were maintained on YPD (2% glucose, 1% yeast extract, 2% bactopeptone and 2% agar) plates at 30 °C. Selection for *S. bombicola* transformants was done with YPD plates supplemented with 500 μg/mL hygromycin B in the case of *S. bombicola ΔSbSLMdr.2,* selection on SD-ura (2% glucose, 0.67% yeast nitrogen base without amino acids (DIFCO), complemented with complete supplement mixture without uracil (MP Biomedicals Europe)) plates was performed for all other *S. bombicola* mutants. *E. coli* transformants were cultivated on Luria-Bertani (LB) (1% tryptone, 0.5% yeast extract, 0.5% NaCl) medium supplemented with 100 mg/L ampicillin. Sophorolipid production on shake flask scale was performed in medium optimized by Lang et al. [59] (132 g/L glucose. H_2_O, 4 g/L yeast extract, 5 g/L Na.citrate.2H_2_O, 1.5 g/L NH_4_Cl, 1 g/L KH_2_PO_4_, 0.16 g/L K_2_HPO_4_, 0.7 g/L MgSO_4_.7H_2_O, 0.5 g/L NaCl, 0.27 g/L CaCl_2_.2H_2_O).

### RNA sequencing

Wild-type *S. bombicola* ATCC 22214 was grown in 100 mL Lang medium without the addition of rapeseed oil at 200 rpm and 30 °C in 500 mL shake flask. 20 mL of culture broth was centrifuged at 4000 g after 24 h, 80 h and 240 h, for isolation of total RNA, representing the exponential, stationary and metabolizing growth phase. Cell pellets were resuspended in 15 mL RNAse-Free water and lyophilized. After crushing the lyophilized residue in liquid nitrogen, the RNA was extracted with the RNeasy Midi kit (Qiagen). Single end, stranded shotgun RNA sequencing was subsequently performed by FASTERIS SA (Switzerland), according to the Illumina library preparation protocol and kit. RNAseq reads were trimmed and quality controlled with FastQC (https://github.com/s-andrews/FastQC). The clean reads were then aligned to the reference genome with Hisat2 [60] and gene expression estimates were reported as raw counts by HTSeq [61]. To identify differentially expressed genes between different experimental conditions, the edgeR package (version 3.6.2) implemented in R was used, only taking into account the genes that have count-per-million (CPM) above 10 in all samples [62]. Multiplicity correction of the results was performed by applying the Benjamini-Hochberg method to control the false discovery rate (FDR). Genes were considered differentially expressed if they possessed an absolute value of log2-fold change ≥ 1 and an adjusted *P*-value ≤ 0.05.

### Cloning and strain engineering

Single gene deletion *S. bombicola* strains were obtained by homologous recombination between the target locus and linear transformation cassette containing the *URA3* selection marker when the *S. bombicola* uracil auxotrophic mutant PT36 was used as background strain. Hygromycin resistance was used to perform genetic modifications in the SLMDR knockout mutant and to knockout *SbSLMdr.2*, with the introduction of an *E. coli* hygromycin resistance gene (*hyg)* codon-harmonized for *S. bombicola* using the Eugene v1.4.0 software based on the *S. bombicola* codon usage table found at https://www.kazusa.or.jp/, and under control of the *S. bombicola* GPD promoter [63]*.* The linear transformation cassettes were created by circular polymerase extension cloning (CPEC) assembly, encompassing the following 4 fragments: pJet vector backbone (Thermo Fischer), ±1000 bp up- and downstream homology regions and the selection marker under control of its endogenous promoter and terminator sequence, amplified from *S. bombicola* genomic DNA. 1 μg of the linear transformation cassette was introduced in *S. bombicola* through electroporation as described previously [64]. The genotype of the transformants was checked by yeast colony PCR All primers used for cloning in this study are listed in Table S4.

### Sophorolipid production, extraction and analysis

5 mL of Lang medium was inoculated with the strains under consideration and incubated at 30 °C and 200 rpm for 48 h. Cells were then transferred to 50 mL fresh Lang medium with or without the addition of 37.5 g/L rapeseed oil in 250 mL sterile shake flasks, where they were incubated for 8 days at 30 °C and 200 rpm. 500 μL samples were taken every 24 h to be analyzed for glucose consumption, OD600, pH and product formation. Sample supernatant was analyzed for the presence of glucose by HPLC as described before [12]. After 8 days of incubation, the production experiment was stopped and sophorolipids extracted by adding 50 mL ethyl acetate. After shaking for 5 min, shake flasks were left to sediment at room temperature for 30 min. The upper fraction was subjected to analysis by UPLC as described in Jezierska et al. [14] Purified wild-type SL mixes were used as external standards for quantification.

### Specific growth rate determination

Specific growth rates for *S. bombicola* strains were determined by incubating 200 μL of cell culture in Lang medium in a flat bottom, 96-well microtiter plate (Greiner Bio-One) at 30 °C in a Tecan Infinite® m200 Pro plate reader (Tecan, Mannedorf, Switzerland) with an initial OD_600_ of 0.05, sealed off with a Breath-Easy® sealing membrane (Sigma-Aldrich). The OD_600_ was measured every 15 min for 40 h, while shaking in between measurements at 200 rpm (orbital mode, amplitude 2 mm). The maximum specific growth rate (μ) was calculated by fitting the Richards model in with the ‘curve_fit ‘function in the ‘SciPy’ module in Python 3.7 [65].

### Statistical analysis

All experiments were performed in triplicates. The results are expressed as the mean values ± standard error of the mean (SE). Statistical differences among groups were found with two-way analysis of variance (ANOVA) with Tukey’s multiple comparisons test using the ‘statsmodels’ module in Python 3.7, unless specified otherwise [66].

### Susceptibility assays

A list of compounds (Table S6) in various concentrations was used to screen for the translocation capacity of putative multidrug exporters in a disc diffusion assay. Strains were grown in 5 mL SD medium for 48 h at 30 °C and 200 rpm. 300 μL of the cell culture (OD_600_ 0.3) was streaked on SD plates (20 mL medium for each plate). A 10 mm paper disk filter impregnated with 60 μL of filter-sterilized tested compound diluted in DMSO was subsequently placed on the plate. After three days of incubation at 30 °C, growth inhibition zones were compared. Susceptibility tests in liquid culture were performed as described for the specific maximum growth rate, with the addition of the filter-sterilized compound to be tested in the concentration as listed in Table S6.

## Supplementary Information

**Additional file 1: Table S1**. Summary table of *S. bombicola*’s predicted transporters. **Table S2**. Keys for TransAAP substrate prediction, grouped. **Table S3**. *S. bombicola* strains used in this research. **Table S4**. Primers used in this research. **Table S5**. Maximum specific growth rate (μ) of selected *S. bombicola* ABC transporter knockouts cultivated on SD medium. **Table S6**. List of compounds used for disc diffusion assay, their concentration in DMSO and supplier. **Figure S1**. Disc diffusion assay with polyoxyethylene-9-lauryl ether of selected *S. bombicola* ABC transporter deletion mutants compared to wild type. **Figure S2**. Disc diffusion assay of *S. bombicola ΔSbCdr4* and *S. bombicola ΔSbYor1.2* compared to wild type. The concentration of the applied medium chain alkanes and fatty alcohols is given in Table S6. **Figure S3**. Comparison of growth among different SL transporter deletion strains on shake flask scale by OD_600_ and pH measurements every 24 h (a) without the addition of rapeseed oil and (b) with the addition of 37.5 g/L rapeseed oil.

## Data Availability

The datasets analyzed during the current study are available in the NCBI Genome repository with the accession number PRJDB2962, https://www.ncbi.nlm.nih.gov/genome/?term=PRJDB2962. Transcriptome data were described before in reference [42], co-authored by the corresponding author, who consequently has personal and administrative access to this, available from the corresponding author upon reasonable request. Transporter sequences analyzed in this study were deposited in GenBank repository for public access under bioproject BankIt2485497: MZ671253-MZ671487.
