# Supplementary material for: Exploring the transportome of the biosurfactant producing yeast Starmerella bombicola
Source: BMC Genomics. 2022 Jan 9;23:22. doi: 10.1186/s12864-021-08177-x (PMC8742932; doi:10.1186/s12864-021-08177-x)
Supplement: Supplementary file 1 — Additional file 1: Table S1. Summary table of S. bombicola’s predicted transporters. Table S2. Keys for TransAAP substrate prediction, grouped. Table S3. S. bombicola strains used in this research. Table S4. Primers used in this research. Table S5. Maximum specific growth rate (μ) of selected S. bombicola ABC transporter knockouts cultivated on SD medium. Table S6. List of compounds used for disc diffusion assay, their concentration in DMSO and supplier. Figure S1. Disc diffusion assay with polyoxyethylene-9-lauryl ether of selected S. bombicola ABC transporter deletion mutants compared to wild type. Figure S2. Disc diffusion assay of S. bombicola ΔSbCdr4 and S. bombicola ΔSbYor1.2 compared to wild type. The concentration of the applied medium chain alkanes and fatty alcohols is given in Table S6. Figure S3. Comparison of growth among different SL transporter deletion strains on shake flask scale by OD600 and pH measurements every 24 h (a) without the addition of rapeseed oil and (b) with the addition of 37.5 g/L rapeseed oil. [file 12864_2021_8177_MOESM1_ESM.docx]

**Table S1** Summary table of *S. bombicola*’s predicted transporters. The UniProt accession number of the closest BLASTp hit is given with the respective e-value, their TCDB classification number (<https://www.tcdb.org/>), the predicted substrate based on the homology, the substrate group according to the trSSP (The Transporter Substrate Specificity Prediction Server (https://www.zhaolab.org/TrSSP/)[1]) and most likely subcellular localization according to DeepLoc prediction combined with experimental evidence of its closest homologue (http://www.cbs.dtu.dk/services/DeepLoc/). SIT: Siderophore-Iron Transporter Family, SP: Sugar Porter Family, MFS: Major Facilitator Superfamily, P-ATPase: P-type ATPase Superfamily, TPPT: Thiamine Pyrosphosphate Transporter Family, YAT: Yeast Amino Acid Transporter Family, DCT: Drug Conjugate Transporter Family, SulP: Sulfate Permease Family, DHA1: Drug:H+ Antiporter-1 (12 Spanner) Family, ATP-E: ATP Exporter Family, ZIP: Zinc-Iron Permease Family, Nramp: Metal Ion (Mn2+-iron) Transporter Family, ENT: Equilibrative Nucleoside Transporter Family, Amt: Ammonium Channel Transporter Family, AAAP: Amino Acid/Auxin Permease Family, ACS: Anion:Cation Symporter Family, YidC/Alb3/Oxa1: Membrane Protein Insertase Family, MC: Mitochondrial Carrier Family, MDR: Multidrug Resistance Exporter Family, CPA1: Monovalent Cation:Proton Antiporter-1 Family, CDF: Cation Diffusion Facilitator Family, NSCC2: Non-selective Cation Channel-2 Family, ClC: Chloride Carrier/Channel Family, OPT: Oligopeptide Transporter Family, AEC: Auxin Efflux Carrier Family, F-ATPase: H+- or Na+-translocating F-type, V-type and A-type ATPase Superfamily, TRP-CC: Transient Receptor Potential Ca2+ Channel Family, PDR: Pleiotropic Drug Resistance Family, EPP: Eye Pigment Precursor Transporter, CaCA: Ca2+:Cation Antiporter Family, SFXN: Sideroflexin Family, KUP: K+ Uptake Permease, SSS: Solute:Sodium Symporter Family, V-BAAT: Vacuolar Basic Amino Acid Transporter, ACT: Amino Acid/Choline Transporter, PHS: Phosphate: H+ Symporter Family, MIT: CorA Metal Ion Transporter Family, Pho1: Phosphate Permease Family, VIC: Voltage-gated Ion Channel Superfamily, CNT: Concentrative Nucleoside Transporter Family, NIPA: NIPA Mg2+ Uptake Permease Family, CitM: Citrate-Mg2+:H+  Citrate-Ca2+:H+ Symporter Family, UMF23: Unidentified Major Faciilitator-23 Family, LAT: L-type Amino Acid Transporter Family, NAG-T: N-Acetylglucosamine Transporter, MPT: Mitochondrial Protein Translocase Family, ArAE: Aromatic Acid Exporter Family, CSA: CMP-Sialate:CMP Antiporter Family, Fluc: Fluoride Channel Family, NAT: Nucleobase/Ascorbate Transporter Family, NCS1: Nucleobase:Cation Symporter-1 Family, LCT: Lysosomal Cystine Transporter Family, Ctr: Copper Transporter Family, MCT: Monocarboxylate Transporter Family, CTL: Choline Transporter Family, EST: Eukaryotic (Putative) Sterol Transporter Family, DHA2: Drug:H+ Antiporter-2 (14 Spanner) Family, PAT: Peptide/Acetyl-Coenzyme A/Drug Transporter Family, LIP/SIP: Lantibiotic Immunity Protein/Serine Connector Protein Family, HMT: Heavy Metal Transporter Family, GPH: Glycoside-Pentoside-Hexuronide:Cation Symporter Family, MATE: Multi Antimicrobial Extrusion Family, TPT: Triose-phosphate Transporter Family, TDT: Tellurite-resistance/Dicarboxylate Transporter Family, CCC: Cation-Chloride Cotransporter Family, POT/PTR: Proton-dependent Oligopeptide transporter Family, OFA: Oxalate:Formate Antiporter Family, UMF12: Unidentified Major Facilitator-12, ABC: ATP-Binding Cassette Superfamily, LTE: Lipid-Translocating Exporter Family, AE: Anion Exchanger Family, MscS: Small Conductance Mechanosensitive Ion Channel Family, APT: Autphagy-related Phagophore-formation Transporter Family, STE: a-Factor Sex Pheromone Exporter Family (ABCB), ThrE: Threonine/Serine Exporter Family, P-FAT: The Peroxisomal Fatty Acyl CoA transporter Family, NCPE: Non-Classical Protein Exporter Family, GPHR: Golgi pH Regulator Family, UGnT: UDP-glucuronate/UDP-N-acetylgalactosamine Transporter Family, GUP: Glycerol Uptake Family, VIT: Vacuolar Iron Transporter Family, PPI2: Integral Membrane Peroxisomal Protein Importer-2 Family, CNNM: Cyclin M Mg2+ Exporter Family, OST: Organic Solute Transporter Family, VBAAT: Vacuolar Basic Amino Acid Transporter, Trk: K+ transporter Family, ILT: Iron/Lead Transporter Family, OLF: Oligosaccharidyl-lipid Flippase Family.

|  |
| --- |

| **SeqId** | **TCDB nr** | **Transporter Family** | **Acc nr BLAST hit** | **e-value** | **Substrate according to homology** | **TransAAP substrate prediction** | **TrSSP substrate prediction** | **Subcellular localization** |
| --- | --- | --- | --- | --- | --- | --- | --- | --- |
| SbEnb1 | 2.A.1.16.2 | SIT | Q08299 | 0 | H+, Fe3+-enterobactin | VI | Cation | Endosome |
| SbStl1.1 | 2.A.1.1.38 | SP | P39932 | 2.2E-51 | glycerol, H+ | VI | Cation | Membrane |
| SbVrtL | 2.A.1 | MFS | D7PHY8 | 2.9E-92 |  | II | Anion | Membrane |
| SbDnf2 | 3.A.3.8.5 | P-ATPase | Q12675 | 0 | Phosphatidylcholine, phosphatidyl serine, Lysophosphoethanolamine | IC | Cation | Cell membrane |
| SbST.1 | 2.A.1 | MFS | O94491 | 2E-115 |  | II | Anion | Endoplasmic reticulum |
| SbST.2 | 2.A.7.24.5 | TPPT | Q03730 | 4.9E-85 |  | VI | Amino Acid | Vacuole |
| SbGap1.1 | 2.A.3.10.2 | YAT | P19145 | 1E-145 | Amino acids | III | Amino Acid | Cell membrane |
| SbGap3.1 | 2.A.3.10.1 | YAT | A0A1D8PN88 | 1.4E-133 | amino acids | III | Amino Acid | Cell membrane |
| SbST.3 | 2.A.1 | MFS | O94343 | 6.6E-124 |  | VI | Amino Acid;Anion | Membrane |
| SbYor1.1 | 3.A.1.208.3 | DCT | P53049 | 0 | multidrug | VI | Sugar | Cell membrane |
| SbSulx | 2.A.53.1.11 | SulP | P53394 | 1.2E-128 | sulfate | ID | Anion;Cation | Endoplasmic reticulum |
| SbFlu1 | 2.A.1.2.16 | DHA1 | G1UB37 | 1.6E-144 | multidrug | VI | Amino Acid | Cell membrane |
| SbGPI7 | 9.A.6.1.1 | ATP-E | Q8TGB2 | 1.4E-170 | ATP | - | Cation | Endoplasmic reticulum |
| SbZrt3 | 2.A.5.5.3 | ZIP | P34240 | 2.1E-23 | Zn2+ | IC | Cation | Vacuole |
| SbSmf1 | 2.A.55.1.1 | Nramp | P38925 | 3.6E-145 | metals | IC | Anion;Cation | Cell membrane |
| SbBtn1 | 2.A.57.5.2 | ENT | Q6BZW3 | 4.9E-133 |  | IV |  | Endoplasmic reticulum |
| SbMep2.1 | 1.A.11.3.2 | Amt | P41948 | 3E-165 | ammonium | IC | Anion;Cation | Cell membrane |
| SbAvt6 | 2.A.18.6.6 | AAAP | P40074 | 4.1E-89 | amino acids | III | Amino Acid | Vacuole |
| SbVht1.1 | 2.A.1.14.19 | ACS | O13880 | 2.8E-110 | Biotin, H+ | II | Amino Acid | Membrane |
| SbOxa1 | 2.A.9.1.1 | YidC/Alb3/Oxa1 | O13375 | 1.2E-90 | proteins | VI | Anion;Proteins/mRNA | Mitochondrial membrane |
| SbMC24 | 2.A.29 | MC | Q54W11 | 5.2E-57 | metabolites, nucleotides, cofactors | - | Anion | Mitochondrial membrane |
| SbPmp | 2.A.29.6.1 | MC | Q00319 | 9.6E-69 |  | - | Amino Acid;Anion;Sugar | Peroxisome membrane |
| SbSLMdr.2 | 3.A.1.201 | MDR | H6TB12 | 0 | sophorolipids | VI | Sugar | Cell membrane |
| SbCrc1 | 2.A.29.8.4 | MC | Q12289 | 2.7E-102 | acetyl-CoA | - | Cation | Mitochondrial membrane |
| SbAgp2 | 2.A.3.10.19 | YAT | P38090 | 7.4E-152 | amino acids | III | Amino Acid | Membrane |
| SbST.4 | 2.A.7.24.5 | TPPT | Q03730 | 5.6E-11 |  | VI | Cation | Vacuole |
| SbST.5 | 2.A.53.3.10 | SulP | P53273 | 0 |  | ID | Anion;Cation | Vacuole |
| SbNhx1 | 2.A.36.1.12 | CPA1 | Q04121 | 0 | Na+, H+ | IC | Amino Acid;Cation | Endosome |
| SbMmt2 | 2.A.4.1.6 | CDF | Q08970 | 3.7E-61 | metal ions | IC | Cation | Mitochondrial membrane |
| SbDrs2 | 3.A.3.8.2 | P-ATPase | P39524 | 0 | Aminophospholipids | IC | Cation | Golgi |
| SbMFS.1 | 2.A.1.1 | SP |  | 5E-56 | sugar | ID | Electron;Sugar | Cell membrane |
| SbST.6 | 2.A.1 | MFS | Q9US44 | 5.5E-64 |  | II | Amino Acid;Sugar | Membrane |
| SbHma5 | 3.A.3.5.3 | P-ATPase | Q9SH30 | 2.1E-175 | copper | IC | Cation | Membrane |
| SbSec62 | 1.A.15.1.1 | NSCC2 | P21825 | 3.7E-66 |  | VI |  | Endoplasmic reticulum |
| SbClcn5 | 2.A.49 | ClC | Q9TTU3 | 1.9E-68 | H+/Cl- | ID | Anion | Golgi |
| SbOpt2.1 | 2.A.67.1.4 | OPT | Q06593 | 8.6E-157 | Glutathione conjugates | III | Anion | Membrane |
| SbST.7 | 2.A.69.2.2 | AEC | P38355 | 3E-130 |  | - |  | Membrane |
| SbVph1.1 | 3.A.2.2.3 | F-ATPase | P32563 | 0 | H+ | IC | Cation | Vacuole |
| SbMFS.2 | 2.A.1.2.16 | DHA1 | G1UB37 | 2.8E-137 | multidrug | VI | Amino Acid;Anion;Sugar | Cell membrane |
| SbIsp4 | 2.A.67.1.2 | OPT | P40900 | 2E-136 | tetrapeptides | III | Anion | Endoplasmic reticulum |
| SbFlc2 | 1.A.4.9.3 | TRP-CC | P39719 | 4.7E-165 | FAD, Heme | IC | Anion | Endoplasmic reticulum |
| SbCtp1 | 2.A.29.7.3 | MC | A0A6M8Y3G7 | 0 | Citrate, Isocitrate |  | Anion;Sugar | Mitochondrial membrane |
| SbCis4 | 2.A.4 | CDF | Q9HGQ3 | 6.8E-71 | cations | IC | Cation | Endoplasmic reticulum |
| SbMFS.3 | 2.A.1.1.39 | SP | B8N0F1 | 3E-73 | glucose | II | Amino Acid | Cell membrane |
| SbCdr4 | 3.A.1.205 | PDR | O74676 | 0 | multidrug | VI |  | Cell membrane |
| SbABCG1 | 3.A.1.204 | EPP | D4AYW0 | 0 | multidrug | VI |  | Endoplasmic reticulum |
| SbVnx1 | 2.A.19.7.1 | CaCA | P42839 | 0 | Na+, K+, H+ | IC |  | Vacuole |
| SbFsf1 | 2.A.54.1.4 | SFXN | Q12029 | 2.2E-117 | Fe3+ | II |  | Mitochondrial membrane |
| SbHak1 | 2.A.72.2.1 | KUP | P50505 | 0 | K+ | IC | Amino Acid;Anion;Sugar | Membrane |
| SbAtp1 | 3.A.2.1.3 | F-ATPase | P07251 | 0 | H+ | IC | Cation;Proteins/mRNA | Mitochondrial membrane |
| SbHgt1.1 | 2.A.1.1.39 | SP | P49374 | 0 | glucose | VI | Amino Acid;Sugar | Cell membrane |
| SbDur31.1 | 2.A.21 | SSS | O94469 | 0 |  | III | Amino Acid;Cation;Sugar | Membrane |
| SbFnx1 | 2.A.1.48.3 | V-BAAT | Q09752 | 1.1E-66 |  | VI |  | Vacuole |
| SbGap1.2 | 2.A.3.10.2 | YAT | P19145 | 0 | Amino acids | III | Amino Acid | Cell membrane |
| SbGap1.3 | 2.A.3.10.2 | YAT | P19145 | 1.8E-168 | Amino acids | III | Amino Acid | Cell membrane |
| SbMFS.4 | 2.A.1.1.39 | SP | B8N0F1 | 8.5E-77 | glucose | II | Amino Acid;Cation;Sugar | Cell membrane |
| SbHgt1.2 | 2.A.1.1.39 | SP | P49374 | 3.8E-126 | glucose | VI | Amino Acid;Cation;Sugar | Cell membrane |
| SbCpaT | 2.A.1 | MFS | F5HN69 | 1.1E-70 |  | VI | Amino Acid;Cation;Sugar | Membrane |
| SbMFS.5 | 2.A.1 | MFS | Q10487 | 4.2E-47 |  | VI | Amino Acid | Endoplasmic reticulum |
| SbBmrp | 2.A.1.2.6 | DHA1 | P28873 | 1.8E-44 | multidrug | VI | Amino Acid;Sugar | Membrane |
| SbHnm1 | 2.A.3.4.1 | ACT | P19807 | 4.3E-60 | choline | III | Amino Acid | Cell membrane |
| SbSeo1 | 2.A.1.14.37 | ACS | P39709 | 0 |  | VI |  | Cell membrane |
| SbUga4.1 | 2.A.3.4.3 | ACT | P32837 | 7.1E-120 | Gamma-aminobutyric acid | III | Amino Acid | Vacuole |
| SbMtp5 | 2.A.4 | CDF | Q5NA18 | 1E-47 | cations | IC | Anion;Cation | Vacuole |
| SbPTH1-2 | 2.A.1.9 | PHS | Q8GSD9 | 2.6E-37 | Phosphate: H+ | VI |  | Membrane |
| SbZntb | 1.A.35.4.2 | MIT | A0A167CNZ2 | 2E-64 | Zn2+, Cd2+ | - |  | Lysosome |
| SbErd1 | 2.A.94.1.3 | Pho1 | P41771 | 3.3E-16 |  | - | Anion;Cation | Endoplasmic reticulum |
| SbIC01 | 1 |  | R9AEG9 | 6.7E-48 |  | II | Electron | Cell membrane |
| SbTok1 | 1.A.1.7.1 | VIC | P40310 | 7.33E-83 | K+ | IC |  | Cell membrane |
| SbST.8 | 2.A.41.2.5 | CNT | Q9UA35 | 7.5E-97 |  | IV |  | Membrane |
| SbNipa4 | 2.A.7.25.4 | NIPA | Q94AH3 | 9.2E-102 |  | VI |  |  |
| SbST.9 | 2.A.11 | CitM | O59712 | 0 |  | II | Anion;Cation | Endoplasmic reticulum |
| SbMep3 | 1.A.11.2 | Amt | P53390 | 8.6E-162 | ammonium | IC | Anion;Cation | Membrane |
| SbMch1.1 | 2.A.1.75.1 | UMF23 | Q96TW9 | 1.5E-39 | monocarboxylate | VI | Amino Acid;Sugar | Vacuole |
| SbVph1.2 | 3.A.2.2.3 | F-ATPase | P32563 | 0 | H+ | IC | Anion | Vacuole |
| SbMup1.1 | 2.A.3.8.4 | LAT | P50276 | 3.7E-158 | L-methionine | III | Amino Acid | Membrane |
| SbMFS.6 | 2.A.1.58.3 | NAG-T | Q9URX1 | 9.2E-36 |  | VI | Amino Acid;Sugar | Membrane |
| SbAlr1.1 | 1.A.35.2.1 | MIT | Q08269 | 3E-121 | Al3+, Mg2+ | IC | Anion;Cation | Cell membrane |
| SbYcf1.1 | 3.A.1.208.11 | DCT | P39109 | 0 | Metal ions, multidrug | VI | Anion | Vacuole |
| SbYia6 | 2.A.29.10.5 | MC | P40556 | 2.6E-74 | NAD+, AMP, GMP | - | Cation | Mitochondrial membrane |
| SbTim17 | 3.A.8.1.1 | MPT | P39515 | 1.1E-67 | proteins | VI | Cation;Electron | Mitochondrial membrane |
| SbSfc1 | 2.A.29.13.1 | MC | P33303 | 1.8E-103 | Succinate, Fumarate | - | Cation;Sugar | Mitochondrial membrane |
| SbST.10 | 2.A.1 | MFS | O43081 | 1.6E-111 |  | VI | Anion;Cation | Membrane |
| SbYcf1.2 | 3.A.1.208.11 | DCT | P39109 | 0 | Metal ions, multidrug | VI | Anion | Vacuole |
| SbMcd4 | 9.A.6.1.1 | ATP-E | Q6C0Z3 | 0 | ATP | - | Anion | Endoplasmic reticulum |
| SbCox18 | 2.A.9.4.1 | YidC/Alb3/Oxa1 | P53239 | 6.9E-40 | proteins | VI | Anion;Proteins/mRNA | Endoplasmic reticulum |
| SbArAe | 2.A.85 | ArAE | A0A2T0FIK2 | 9E-161 | aromatic acids | VI |  | Cell membrane |
| SbST.11 | 2.A.7.12.13 | CSA | O59785 | 1.8E-115 |  | VI | Cation | Golgi |
| SbAvt3 | 2.A.18.7.3 | AAAP | Q10074 | 5.6E-155 | amino acids | III | Anion | Endoplasmic reticulum |
| SbFex1 | 1.A.43.2.4 | Fluc | Q5AFH3 | 2.9E-31 | Fl-, Cl- | - | Anion | Cell membrane |
| SbYI31 | 2.A.40 | NAT | Q9HE12 | 0 | purine | IV | Anion | Vacuole |
| SbNeo1 | 3.A.3.8.18 | P-ATPase | P40527 | 0 | phospholipids | IC | Anion | Endoplasmic reticulum |
| SbSyg1 | 2.A.94 | Pho1 | P40528 | 1.2E-78 |  | - |  | Cell membrane |
| SbDur3 | 2.A.21.6.1 | SSS | P33413 | 0 | Urea, Polyamines | III | Amino Acid | Cell membrane |
| SbSit1 | 2.A.1 | MFS | Q4WGS5 | 8.7E-161 | ferichrome, ferrioxamine | VI | Amino Acid;Anion | Cell membrane |
| SbAlr1.2 | 1.A.35.2.1 | MIT | Q08269 | 4E-160 | Al3+, Mg2+ | IC |  | Cell membrane |
| SbCch1 | 1.A.1.11.23 | VIC | O14234 | 6.3E-178 | Ca2+ | IC | Anion | Cell membrane |
| SbFcy21 | 2.A.39 | NCS1 | P40039 | 2.7E-130 | purine | V |  | Cell membrane |
| SbMch1.2 | 2.A.1.75.1 | UMF23 | Q4IM48 | 3.4E-18 | monocarboxylate | VI | Amino Acid;Anion | Vacuole |
| SbItr2 | 2.A.1.1.104 | SP | P30606 | 1.9E-156 | myoinositol | VI | Sugar | Cell membrane |
| SbYbt1 | 3.A.1.208.12 | DCT | P32386 | 0 | bile acids | VI | Anion | Vacuole |
| SbMFS.7 | 2.A.1 | MFS | A0A2T0FEV1 | 9E-106 |  | VI |  | Cell membrane |
| SbST.12 | 2.A.1.1.96 | SP | P38142 | 4.5E-54 | metabolites | VI | Anion;Sugar | Vacuole |
| SbYpq3 | 2.A.43.2.7 | LCT | P38279 | 1.4E-41 | cationic amino acids | - |  | Vacuole |
| SbCtr3 | 1.A.56.1.4 | Ctr | Q06686 | 2.3E-23 | Cu+ | IC | Cation | Membrane |
| SbHol1 | 2.A.1.2.33 | DHA1 | P53389 | 1.5E-62 |  | VI | Amino Acid;Anion | Membrane |
| SbAvt2 | 2.A.18.6.20 | AAAP | P39981 | 7.9E-128 | amino acids | III | Amino Acid;Anion | Vacuole |
| SbStl1.2 | 2.A.1.1.38 | SP | P39932 | 6.3E-80 | glycerol, H+ | VI | Amino Acid;Sugar | Membrane |
| SbMFS.8 | 2.A.1.13 | MCT | B8N0F1 | 1.3E-87 | monocarboxylate | VI | Amino Acid;Anion | Cell membrane |
| SbST.13 | 2.A.1.14.38 | ACS | P40445 | 2.2E-135 | Membrane | II |  |  |
| SbOac1 | 2.A.29.15.1 | MC | P32332 | 4.4E-103 | oxaloacetate, Malonate, Sulfate, Thiosulfate | - | Anion;Sugar | Mitochondrial membrane |
| SbPns1 | 2.A.92.1.4 | CTL | Q870V7 | 1.3E-42 | choline | - | Amino Acid;Sugar | Cell membrane |
| SbMFS.9 | 2.A.1 | MFS | Q4WC50 | 4.3E-45 |  | VI | Anion;Sugar | Membrane |
| SbAgp3 | 2.A.3.10.14 | YAT | P43548 | 1E-177 | amino acids | III | Amino Acid;Sugar | Membrane |
| SbST.14 | 2.A.67.1 | OPT | Q7LL00 | 0 | oligopeptide | III | Amino Acid;Sugar | Membrane |
| SbDal5 | 2.A.1.14.4 | ACS | P15365 | 1.6E-118 | dipeptides | II | Anion;Sugar | Membrane |
| SbGit1.1 | 2.A.1.9.7 | PHS | A0A3F2XXJ6 | 1E-138 | glycerophosphoinositol, glycerophosphocholine | VI | Amino Acid;Anion;Sugar | Cell membrane |
| SbNpc1 | 2.A.6.6.1 | EST | O15118 | 3.8E-162 | cholesterol, lipids | VI | Anion;Cation | Vacuole |
| SbDur31.2 | 2.A.21 | SSS | O94469 | 0 | urea | III | Amino Acid;Anion;Cation | Membrane |
| SbYcf1.3 | 3.A.1.208.11 | DCT | P39109 | 0 | Metal ions, multidrug | VI | Anion | Vacuole |
| SbVma5 | 3.A.2.2.3 | F-ATPase | P31412 | 1.7E-81 | H+ | IC |  | Vacuole |
| SbAtr1 | 2.A.1.3.1 | DHA2 | P13090 | 9.4E-112 | Aminotriazole, H+, L-cysteine, 4-nitroquinoline-N-oxide | III | Amino Acid | Membrane |
| SbST.15 | 2.A.1.25.4 | PAT | P38318 | 1.7E-146 | Peptide, Acetyl-CoA | VI | Anion | Membrane |
| SbTms1 | 9.A.29.3.4 | LIP/SIP | Q12116 | 1.7E-136 |  | - |  | Membrane |
| SbHgt1.3 | 2.A.1.1.39 | SP | P49374 | 7.9E-126 | glucose | VI | Amino Acid;Anion;Sugar | Cell membrane |
| SbAtm1 | 3.A.1.210 | HMT | Q6CX96 | 0 |  | V | Proteins/mRNA;Sugar | Mitochondrial membrane |
| SbZIP9 | 2.A.5.6.1 | ZIP | Q5ZIU9 | 1.1E-23 | Zn2+ | IC | Amino Acid;Cation | Membrane |
| SbCopA | 3.A.3 | P-ATPase | Q4A0G1 | 2.3E-136 | Cu+ | IC | Cation | Cell membrane |
| SbOpt2.2 | 2.A.67.1.4 | OPT | Q06593 | 0 | Glutathione conjugates | III | Anion | Cell membrane |
| SbStl1.3 | 2.A.1.1.38 | SP | P39932 | 0 | glycerol, H+ | VI | Anion;Sugar | Cell membrane |
| SbVma3 | 3.A.2.2.3 | F-ATPase | P50515 | 3.6E-87 | H+ | IC | Cation | Vacuole |
| SbFus6 | 2.A.1.2.68 | DHA1 | W7MLD3 | 4.1E-73 | tetracycline, multidrug | VI | Amino Acid;Anion | Membrane |
| SbErs1 | 2.A.43.1.3 | LCT | XP_024663938.1 | 6E-100 |  | - |  | Vacuole |
| SbSut1 | 2.A.2.6.1 | GPH | O14091 | 8.6E-44 | Maltose, Sucrose, H+ | II | Cation | Membrane |
| SbDnf1 | 3.A.3.8.4 | P-ATPase | P32660 | 0 | phospholipids | IC | Cation;Electron | Cell membrane |
| SbErc1 | 2.A.66.1.5 | MATE | P38767 | 4.1E-112 | multidrug | VI | Anion;Cation | Membrane |
| SbST.16 | 2.A.3.10.21 | YAT | O60113 | 3.2E-170 | amino acids | III | Amino Acid;Anion | Membrane |
| SbItr1 | 2.A.1.1.104 | SP | P87110 | 5.6E-47 | myoinositol | VI | Amino Acid;Anion;Sugar | Cell membrane |
| SbMdr2 | 3.A.1.201 | MDR | Q4WPP6 | 0 | multidrug | VI | Proteins/mRNA;Sugar | Membrane |
| SbST.17 | 2.A.67.2.7 | OPT | P53134 | 3.9E-173 | oligopeptide | III | Anion | Membrane |
| SbMFS.10 | 2.A.7.9.16 | TPT | Q10354 | 1.2E-53 |  | VI | Amino Acid;Sugar | Membrane |
| SbST.18 | 2.A.19 | CaCA | P87122 | 6.3E-50 | Ca2+/cation | IC | Anion;Cation | Endoplasmic reticulum |
| SbGit1.2 | 2.A.1.9.7 | PHS | Q59Q30 | 7.4E-165 | Glycerophosphoinositol, glycerophosphocholine | VI | Amino Acid;Anion;Sugar | Cell membrane |
| SbMae1 | 2.A.16.2.1 | TDT | P50537 | 1.4E-66 | malate | ID | Anion;Electron;Sugar | Membrane |
| SbLiz1 | 2.A.1.14.17 | ACS | O43000 | 2.2E-80 | H+, Pantothenate | II | Amino Acid | Cell membrane |
| SbVhc1 | 2.A.30.1.2 | CCC | P38329 | 0 | NaCl, KCl | III | Anion;Cation | Vacuole |
| SbGap3.2 | 2.A.3.10.1 | YAT | A0A1D8PN88 | 2E-130 | Amino acids | III | Amino Acid;Anion | Cell membrane |
| SbPtr2 | 2.A.17.2.2 | POT/PTR | P32901 | 5.9E-152 | peptides, H+ | III | Amino Acid;Anion | Membrane |
| SbZrc1 | 2.A.4.2.2 | CDF | P20107 | 1.3E-116 | Zn2+, Cd2+, H+ | IC | Anion;Cation | Mitochondrial membrane |
| SbTom20 | 3.A.8.1.1 | MPT | P35180 | 7.4E-34 | proteins | VI | Proteins/mRNA | Mitochondrial membrane |
| SbFur4 | 2.A.39.3.2 | NCS1 | P05316 | 6.3E-155 | Uracil, Uridine | V |  | Membrane |
| SbYPR011 | 2.A.29.23.9 | MC | Q12251 | 2.1E-73 | PAPS, APS, phosphate, Sulfate | - | Anion;Sugar | Mitochondrial membrane |
| SbVma11 | 3.A.2.2.3 | F-ATPase | Q6BSB9 | 1.4E-74 | H+ | IC | Anion;Cation | Vacuole |
| SbCyhr.1 | 2.A.1.2.2 | DHA1 | P32071 | 2.3E-130 | cycloheximide, H+ | VI | Amino Acid;Anion;Sugar | Membrane |
| SbYor1.2 | 3.A.1.208.3 | DCT | P53049 | 0 | multidrug | VI |  | Cell membrane |
| SbAnt1 | 2.A.29.17.1 | MC | Q06497 | 1.2E-68 | ATP, ADP, AMP | - | Amino Acid;Anion | Peroxisome membrane |
| SbMFS.11 | 2.A.1.2.16 | DHA1 | G1UB37 | 2.2E-136 | multidrug | VI | Amino Acid;Sugar | Cell membrane |
| SbAtc2 | 3.A.3.2.2 | P-ATPase | P38929 | 0 | Ca2+ | IC | Anion;Sugar | Vacuole |
| SbGms1 | 2.A.7.12.12 | CSA | P87041 | 2.7E-93 | UDP-sugar | IV | Cation;Sugar | Golgi |
| SbNah2 | 2.A.36.4.5 | CPA1 | O14123 | 0 | Na+, H+ | IC | Cation;Electron | Membrane |
| SbST.19 | 2.A.1.11.4 | OFA | P46996 | 0.000084 |  | VI |  | Membrane |
| SbSLMdr.1 | 3.A.1.201 | MDR | H6TB12 | 0 | sophorolipids | VI | Sugar | Cell membrane |
| SbST.20 | 2.A.1.63.4 | UMF12 | P47159 | 9.6E-82 |  | VI | Amino Acid;Sugar | Membrane |
| SbAgc1 | 2.A.29 | MC | Q9VA73 | 1.7E-163 | metabolites, nucleotides, and cofactors | - |  | Mitochondrial membrane |
| SbLpe10 | 1.A.35 | MIT | Q6C8H7 | 5.8E-111 | metal ion | IC |  | Mitochondrial membrane |
| SbTim10 | 3.A.8.1.1 | MPT | Q6C6U1 | 3.1E-29 | proteins | VI | Electron;Proteins/mRNA | Mitochondrial membrane |
| SbPic2 | 2.A.29.4.4 | MC | P40035 | 2.4E-131 | phosphate | - | Amino Acid;Anion | Mitochondrial membrane |
| SbABC.1 | 3.A.1 | ABC | Q12298 | 4.5E-80 |  | V | Proteins/mRNA | Mitochondrial membrane |
| SbYB8B | 2.A.29 | MC | O59674 | 7.1E-43 |  | - |  | Mitochondrial membrane |
| SbZPT71 | 2.A.5 | ZIP | A8WMY3 | 1.8E-37 | metal ion | IC | Anion;Cation | Membrane |
| SbOpt2.3 | 2.A.67.1.4 | OPT | Q06593 | 2.3E-149 | Glutathione conjugates | III | Anion | Membrane |
| SbAmt2 | 1.A.11.2 | Amt | Q9US00 | 7.3E-68 | ammonium | IC | Anion;Cation | Membrane |
| SbYhm2 | 2.A.29.29.1 | MC | Q04013 | 7.6E-129 | Citrate, Succinate, Fumarate | - | Sugar | Mitochondrial membrane |
| SbST.21 | 2.A.7.9.18 | TPT | O94695 | 1.5E-70 |  | II | Amino Acid;Anion;Sugar | Membrane |
| SbST.22 | 2.A.1 | MFS | Q9USN4 | 4.2E-100 |  | VI |  | Membrane |
| SbST.23 | 2.A.1 | MFS | O43081 | 7.3E-139 |  | VI | Amino Acid;Sugar | Membrane |
| SbRsb1 | 9.A.26.1 | LTE | C8ZI10 | 6.1E-43 | sphingoid long-chain bases | - | Amino Acid | Cell membrane |
| SbVma6 | 3.A.2.2.3 | F-ATPase | P32366 | 5.7E-137 | H+ | IC | Cation;Proteins/mRNA | Vacuole |
| SbFlc3 | 1.A.4.9.3 | TRP-CC | P53121 | 1.3E-128 | FAD, Heme | IC | Anion | Endoplasmic reticulum |
| SbSpf1 | 3.A.3.10.3 | P-ATPase | P39986 | 0 | Mn2+ | IC | Anion | Endoplasmic reticulum |
| SbBor1 | 2.A.31.3.2 | AE | P53838 | 6.7E-162 | boric acid | ID | Amino Acid;Anion;Sugar | Cell membrane |
| SbTpc1 | 2.A.29.28.1 | MC | A7ER02 | 6.7E-65 | thiamine pyrophosphate | - | Sugar | Mitochondrial membrane |
| SbMsy2 | 1.A.23 | MscS | O14050 | 4.2E-79 | osmolytes | IA | Proteins/mRNA | Endoplasmic reticulum |
| SpYpq2.1 | 2.A.43.2.8 | LCT | Q06328 | 7.9E-29 | amino acids | - |  | Cell membrane |
| SbZrg17 | 2.A.4.4.1 | CDF | P53735 | 3E-96 | Zn2+ | IC |  | Endoplasmic reticulum |
| SbYpk9 | 3.A.3.10.8 | P-ATPase | Q12697 | 0 | metal ion | - | Cation | Vacuole |
| SbAtg9 | 9.A.15.1.1 | APT | A7KAM0 | 2.8E-165 | phospholipids | III |  | Lysosome |
| SbHst6 | 3.A.1.206 | STE | P53706 | 1.7E-83 | alpha-factor | VI | Anion;Sugar | Membrane |
| SbMC33 | 2.A.29 | MC | Q20799 | 0.000022 | glutamate/aspartate | - | Anion;Sugar | Mitochondrial membrane |
| SbStl1.4 | 2.A.1.1.38 | SP | P39932 | 0 | glycerol, H+ | VI | Anion;Sugar | Membrane |
| SbAbcc1 | 3.A.1.208 | DCT | O35379 | 4.7E-117 | multidrug | VI |  | Cell membrane |
| SbCtr2 | 1.A.56.1.3 | Ctr | P38865 | 2.3E-30 | Cu+ | IC | Anion;Cation | Vacuole |
| SbMFS.12 | 2.A.1 | MFS | A0A316W8Z8 | 4E-135 |  | VI | Amino Acid;Sugar | Cell membrane |
| SbTpn1 | 2.A.39.2.2 | NCS1 | P53099 | 2E-76 | Vitamin B6, H+, Pyridoxine, Pyridoxal, Pyridoxamine | V | Amino Acid;Anion | Cell membrane |
| SbPrm10 | 2.A.79.1.10 | ThrE | Q6C4Q0 | 0 |  | - |  | Membrane |
| SbGap3.3 | 2.A.3.10.1 | YAT | A0A1D8PN88 | 8.9E-147 | Amino acids | III | Amino Acid | Cell membrane |
| SbPxa2 | 3.A.1.203.6 | P-FAT | P34230 | 1.5E-166 | long-chain fatty acids, Oleic acid | VI | Proteins/mRNA | Peroxisome membrane |
| SbNce2 | 9.A.27.1.1 | NCPE | Q12207 | 1.4E-28 | proteins | - |  | Cell membrane |
| sbMrs2 | 1.A.35 | MIT | Q6FV22 | 3.5E-67 | metal ion | IC |  | Mitochondrial membrane |
| SbMFS.13 | 2.A.1.2.16 | DHA1 | G1UB37 | 6.9E-133 | multidrug | VI | Amino Acid;Anion;Sugar | Cell membrane |
| SbHut1 | 2.A.7.12 | CSA | Q6C4X5 | 1.7E-117 | UDP-sugar | VI | Anion;Sugar | Endoplasmic reticulum |
| SbRtc2 | 2.A.43.2.7 | LCT | P38279 | 2.8E-69 | cationic amino acids | - | Anion | Vacuole |
| SbCmc1 | 2.A.29.23.2 | MC | P0CI40 | 4.5E-125 | ATP | - | Sugar | Mitochondrial membrane |
| SbGphrb | 1.A.38 | GPHR | B7ZAQ6 | 5.9E-15 |  | - | Anion;Cation | Endoplasmic reticulum |
| SbVrg4 | 2.A.7.15.3 | UGnT | Q6C0U0 | 1.1E-138 | UDP-sugar | II | Amino Acid;Sugar | Golgi |
| SbYor1.3 | 3.A.1.208.3 | DCT | P53049 | 7.6E-177 | multidrug | VI | Anion;Cation | Cell membrane |
| SbZrt1 | 2.A.5.1.1 | ZIP | P32804 | 2E-141 | Zn2+, Cd2+ | IC | Amino Acid;Anion;Cation | Membrane |
| SbUga4.2 | 2.A.3.4.3 | ACT | P32837 | 2.4E-117 | Gamma-aminobutyric acid | III | Amino Acid;Anion;Sugar | Cell membrane |
| SbVma16 | 3.A.2.2.3 | F-ATPase | O14046 | 3E-72 | H+ | IC | Anion | Vacuole |
| SbCyhr.2 | 2.A.1.2.2 | DHA1 | P32071 | 5E-153 | cycloheximide, H+ | VI | Amino Acid | Membrane |
| SbGup1 | 2.A.50.1.1 | GUP | P53154 | 4.7E-171 | glycerol, H+ | II |  | Membrane |
| SbTim22 | 3.A.8.1.1 | MPT | A0A1D8PI78 | 2E-73 | proteins | VI |  | Mitochondrial membrane |
| SbFfz1 | 2.A.1.2.23 | DHA1 | ALK02043.1 | 0 | Fructos e | VI | Amino Acid;Anion;Sugar | Cell membrane |
| SbBat1 | 2.A.3.8.5 | LAT | A0A2T0FJ19 | 8.64E-94 | Cystine, glutamate | VI | Amino Acid | Cell membrane |
| SbCcc1 | 2.A.89.1.1 | VIT | P47818 | 6.8E-64 | Fe2+, Mn2+ | IC | Amino Acid;Cation | Vacuole |
| SbMnr2 | 1.A.35.2.2 | MIT | A0A2T0FCE2 | 4.2E-151 | metal ions | IC |  | Membrane |
| SbPex3 | 9.A.17.1.2 | PPI2 | Q874C0 | 9.7E-78 | proteins | IC | Proteins/mRNA | Peroxisome membrane |
| SbTim21 | 3.A.8.1.1 | MPT | Q6CW96 | 8.1E-35 | proteins | VI | Anion;Electron | Mitochondrial membrane |
| SbMam3 | 1.A.112.1.6 | CNNM | Q12296 | 9.4E-180 | metal ions | VI |  | Cell membrane |
| SbST.24 | 2.A.1.63.4 | UMF12 | P47159 | 1.7E-82 |  | VI | Anion;Sugar | Membrane |
| SbPma1 | 3.A.3 | P-ATPase | P28877 | 0 | H+ | IC | Cation | Cell membrane |
| SbPlt5 | 2.A.1.1.34 | SP | Q8VZ80 | 5.8E-45 | myo-inositol, glycerol, Ribose, Sorbitol, mannitol, Xylitol, Erythritol, H+ | VI | Anion;Cation | Cell membrane |
| SbAdt2 | 2.A.29.1.7 | MC | P18239 | 4E-145 | ATP, ADP | - | Amino Acid;Anion;Sugar | Mitochondrial membrane |
| SbDnf3 | 3.A.3.8.20 | P-ATPase | Q12674 | 0 | phospholipid | IC | Cation;Proteins/mRNA | Cell membrane |
| SbTm184 | 2.A.82.1.8 | OST | Q09906 | 2.4E-71 |  | - |  | Endoplasmic reticulum |
| SbMtr.1 | 2.A.18.4.1 | AAAP | P38680 | 4.3E-71 | amino acids | III | Anion | Cell membrane |
| SbYpq2.2 | 2.A.43.2.8 | LCT | Q06328 | 1.1E-16 | amino acids | - |  | Cell membrane |
| SbVcx1 | 2.A.19.2.2 | CaCA | Q99385 | 1.1E-130 | Ca2+ | IC | Amino Acid;Cation | Vacuole |
| SbMtr.2 | 2.A.18.4.1 | AAAP | P38680 | 5.8E-10 | amino acids | III |  | Cell membrane |
| SbST.25 | 2.A.82.1.8 | OST | O94343 | 3.2E-160 |  | VI | Amino Acid;Anion | Vacuole |
| SbDbad | 2.A.1.13 | MCT | Q5AUY2 | 1.8E-68 | monocarboxylate | VI | Amino Acid;Sugar | Cell membrane |
| SbSmf2 | 2.A.55.1.2 | Nramp | P38778 | 2.5E-171 | Mn2+, Cu2+ | IC | Amino Acid;Cation | Membrane |
| SbYvc1 | 1.A.4.4.1 | TRP-CC | Q5A2J7 | 3.6E-08 | Ca2+ | IC |  | Membrane |
| SbAtg22 | 2.A.1.24.2 | VBAAT | A7KAK4 | 6.1E-127 | Arginine, lysine, Histidine | VI | Anion;Cation | Membrane |
| SbPxa1 | 3.A.1.203.6 | P-FAT | P41909 | 0 | long-chain fatty acids, Oleic acid | VI | Proteins/mRNA | Peroxisome membrane |
| SbFun26 | 2.A.57.3.1 | ENT | P31381 | 5.3E-34 | Uridine, Adenosine, Cytidine | IV | Amino Acid;Sugar | Membrane |
| SbST.26 | 2.A.1.3.69 | DHA2 | Q03263 | 1.4E-124 | multidrug | III | Amino Acid;Sugar | Membrane |
| SbMup1.2 | 2.A.3.8.4 | LAT | P50276 | 1E-70 | L-methionine, selenomethionine | III | Amino Acid | Membrane |
| SbVht1.2 | 2.A.1.14.19 | ACS | O13880 | 4E-128 | Biotin, H+ | II | Amino Acid | Membrane |
| SbMFS.14 | 2.A.1 | MFS | Q4WC50 | 1.1E-44 |  | VI | Sugar | Cell membrane |
| SbST.27 | 2.A.1.11.4 | OFA | P46996 | 8.7E-16 |  | VI | Amino Acid | Membrane |
| SbSul2 | 2.A.53.1.13 | SulP | Q12325 | 0 | sulfate | ID | Anion | Vacuole |
| SbTrk2 | 2.A.38.2.3 | Trk | P28584 | 2.5E-140 | K+ | IC |  | Membrane |
| SbFtra | 2.A.108.1.4 | ILT | E9QT42 | 3.6E-111 | Iron 2+, Iron 3+ | IC | Anion;Cation | Membrane |
| SbQdr2 | 2.A.1.2.43 | DHA1 | Q59YT1 | 2.1E-85 | multidrug | VI | Amino Acid;Sugar | Cell membrane |
| SbNha1 | 2.A.36.4.1 | CPA1 | Q99271 | 1.1E-130 | Na+, K+, H+ | IC | Sugar | Cell membrane |
| SbRft1 | 2.A.66.3.1﻿ | OLF | Q6C6S3 | 1.3E-67 | sugar | VI |  | Membrane |
| SbPfmaC | 2.A.1 | MFS | W3X9K4 | 1.9E-64 |  | II | Anion | Cell membrane |
| SbPmr1 | 3.A.3.2.3 | P-ATPase | P13586 | 0 | Ca2+, Cd2+, Mn2+ | IC | Proteins/mRNA;Sugar | Membrane |
| SbST.28 | 2.A.49 | ClC | O60159 | 1.2E-139 | H+/Cl- | ID | Anion;Cation | Membrane |
| SbAtp3 | 3.A.2.1.3 | F-ATPase | Q6C338 | 4.6E-122 | H+ | IC | Proteins/mRNA | Mitochondrial membrane |
| SbGit4 | 2.A.1.1 | SP | A0A1D8PN14 | 5E-145 | Glycerophosphocholine | VI | Amino Acid;Sugar | Membrane |
| SbMep2.2 | 1.A.11.3.2 | Amt | P41948 | 4E-116 | ammonium | IC | Amino Acid;Anion;Cation | Cell membrane |
| SbST.29 | 2.A.3.8.5 | LAT | O60113 | 4.6E-149 | amino acids | III | Amino Acid;Anion;Sugar | Membrane |

**Table S2** Keys for TransAAP substrate prediction, grouped

| **Substrate Group** | **Substrates** |
| --- | --- |
| Amino acids | amino acid, amine, amide, amidate, polyamine, peptide, pheromone, BacA, SbmA, Val, Ile, cys, alanine, GABA, aminobutyrate, arginine, asparagine, aspartate, cystine, cysteine, glutamate, glutathione, GlnQ, glycine, histidine, serine, leucine, lysine, methionine, ornithine, proline, threonine, tryptophan, tyrosine, valine, betaine, carnitine, choline, neurotransmitter, putrescine taurine, serotonin, urea, Cystinosin, sl15, quaternary ammonium, spermidine, SugE, ISP4, Sexual differentiation, P-protein, organic cation |
| Multidrug | aminotriazole, daunorubicin, microcin, bile, lipids, acriflavin, bicyclomycin, bleomycin, chloramphenicol, cycloheximide, erythromycin, bromide, fosmidomycin, kanamycin, macrolide, puromycin, methlenomycin, streptomycin, tetracyclin, toluene, toulene, steroid, hormone, methylviologen, acridine, respiration, ALD, bacitracin, Bcl-2, glucan, colicin, cyclolysin, DinF, efflux, export, ethanolamin, heterocyst, hemolysin, lincomycin, polysaccharide, lantibiotic, viologen, MmpL MviN, enzyme, nodulation, O-antigen, oxidase, lipid, lipoprotein, protease, RarD, salivaricin, Sec61, secretion, antigen, teichoic, toxin, vanadate, antimicrobics, bacteriocin, erythromycin, SecDF, Brefeldin, sterol, quinolone, lactococcin |
| Anion | chloride, Cl, CO2, HCO3, cyanate, sulfate, sulphate, sulfonate, tellurite, arsenite, arsenic, phosphate, Pi, phosphonate, iodide, chromate, biocarbonate, nitrate, nitrite, anion, molybd, molydbenum, antimonite, sulfonate, tungst, silicate, silicon |
| Cation | sodium, potassium, K+, Na, H+, Ca2, Cu2+, Mg, Mn2, NH4, Zn2, Al3, Fe2, Fe(II), proton IDzinc, manganese, calcium, magnesium, cobalt, cation, ferric, ammonium, CD20, cadmium, chelated, copper, cyclic nucleotide gated, metal, iron, lead, mercur, metal, nickel, Polycystin, vanilloid, ferrous, MRS |
| Vitamin | vitamin, ascorbate, biotin, folate, biopterin, nicotinate, thiamin, pantothenate, coenzyme, CoA, GDP, UDP, CMP, (III), iron compound, enterobactin, siderophore, pyoverdine, ferrichrome, vibriobactin, prostaglandin, Cyd, Cyc, cytochrom, AmpG, heme, pigment, hemin, cell division, FLVCR, panthothenate, SpoIIIJ, Oxa1, YidC, pteridine, Fe-S |
| Nucleotides | Allantoin, cytosine, nucleobase, uracil, purine, xanthine, nucleoside, nucleotide, uridine, ADP, pyrimidine, ribonucleotide, xanthosine, FAD |
| Sugar | sugar, carbohydrate, carboxyl, saccharide, cellobiose, trehalose, ribose, fructose, fucose, galactitol, galactoside, galactose, [Gg]lucose, [Hh]exose, hexuronate, lactose, maltose, melibiose, mannose mannitol, arabinose, AraJ, arbutin, myoinositol, raffinose, glucitol, sorbitol, rhamnose, sialic acid, sucrose, xylose, acetate, acetic, auxin, benzoate, formate, galacturonate, propionate, gluconate, glucoside, hexuronate, lactate, muconate, muconolactone, phthalate, pyruvate, shikimate, tartrate, citrate, malate, malonate, oxalate, triose, Aga, Sga, glucoside, glutarate, glucarate, glucuronide, gluterate, phosphoglycerate, [Gg]lycerol, arabinose, malic acid, mannitol, maltodextrin, melibiose, metabolite, nitrogen regula, nicotinic, pentitol, rhizopine, solute, sorbitol, sorbose, inositol, MadN, allantoate, fatty acid, arabinitol, galactarate, vanillate, organic anion, glycerol, glucose, uncoupling |

**Table S3** *S. bombicola* strains used in this research

| **Name** | **Genotype** | **Reference** |
| --- | --- | --- |
| *S. bombicola* ATCC 22214 | Wild type | ATCC 22214 |
| *S. bombicola* PT36 | *ΔURA3* | [2] |
| *S. bombicola ΔSLMdr.1* | *SLMdr.1::URA3* | [3] |
| *S. bombicola ΔCdr4* | *SbCdr4::URA3* | This research |
| *S. bombicola ΔYor1.1* | *SbYor1.1::URA3* | This research |
| *S. bombicola ΔYcf1.2* | *SbYcf1.2::URA3* | This research |
| *S. bombicola ΔYor1.2* | *SbYor1.2::URA3* | This research |
| *S. bombicola ΔSLMdr.2* | *SLMdr.2::hyg* | This research |
| *S. bombicola ΔSLMdr.1ΔSLMdr.2* | *SLMdr.1::URA3,*  *SLMdr.2::hyg* | This research |

**Table S4** Primers used in this research

| **Primer** | **Sequence** |
| --- | --- |
| P2333_07510down_gib_ura3_R | GCTGAGAATATTGTAGGAGATCTTCTAGAAAGATCAACCAGGTTGGACTCTGTAG |
| P2334_07510down_gib_ura3_F | TGGAGTTGATGACGATGTGTATAGTGACGATTTCTTCGCTCAATACCAAACC |
| P2335_07510up_gib_pJet_F | GCTCGAGTTTTTCAGCAAGATCATCTCACGGACTAGGGTGATATTC |
| P2336_07510up_gib_ura3_R | GCCATCATGGTTCAACCTCACTCAGCTTGCCAATTCCAAATTATCC |
| P2337_ura3_gib_07510up_F | GAATTGGCAAGCTGAGTGAGGTTGAACCATGATGGCAGTGTTC |
| P2338_ura3_gib_07510down_R | GGTTTGGTATTGAGCGAAGAAATCGTCACTATACACATCGTCATCAACTC |
| P2339_pJet_07510up_R | CCATGAATATCACCCTAGTCCGTGAGATGATCTTGCTGAAAAACTCGAGCCATC |
| P2340_pJet_07510down_F | ACAGAGTCCAACCTGGTTGATCTTTCTAGAAGATCTCCTACAATATTC |
| P2431_07510_KO_cassette_R | CAACCAGGTTGGACTCTGTAG |
| P2432_07510_KO_cassette_F | CATCTCACGGACTAGGGTGATATTC |
| P2442_5'region_07510_F | ATAAGGCGTCCTGGATTGGTTG |
| P2443_3'region_07510_R | CATGGTGGAGCTGAAACTGGAAAC |
| P2231_00980up_gib_pJet_F | AGATCTTCCGGATGGCTCGAGTTTTTCAGCAAGATAGGTCTCACGCTCACACTC |
| P2326_00980up_gib_ura3_R | CGAACACTGCCATCATGGTTCAACCTCACTATTTGTGTGAAGTCGACATAATTC |
| P2327_00980down_gib_ura3_F | GTTGATGACGATGTGTATAGTGACGATGACTATGACTCCCTGCCTCAATTACC |
| P2236_00980down_gib_pJet_R | CTGAGAATATTGTAGGAGATCTTCTAGAAAGATTTATTGCCGGAACTGGATGCA  AGCTC |
| P2328_ura3_gib_00980_R | GAGGCAGGGAGTCATAGTCATCGTCACTATACACATCGTCATCAACTC |
| P2329_ura3_gib_00980_F | GTCGACTTCACACAAATAGTGAGGTTGAACCATGATGGCAGTGTTC |
| P2229_pJet_gib_00980up_R | GAGTGTGAGCGTGAGACCTATCTTGCTGAAAAACTCGAGCCATCCGGAAGATC |
| P2230_pJet_gib_00980down_F | GTGAGCTTGCATCCAGTTCCGGCAATAAATCTTTCTAGAAGATCTCCTACAATA  TTC |
| P2429_00980_KO_cassette_R | TTATTGCCGGAACTGGATGCAAGCTC |
| P2441_00980_KO_cassette_F | TAGGTCTCACGCTCACACTC |
| P2449_3'region_00980_R | GGGCTTCAGCCAGCTAAGTG |
| P2450_5'region_00980_F | GCCGTTTGAGACTCATGCTG |
| P2512_ura3_gib_13050up_F | TTCTGTGCGCTCTCGGTGAGGTTGAACCATGATGGCAGTGTTC |
| P2513_ura3_gib_13050down_R | GCTCCTAGCAGCAGATCGTCACTATACACATCGTCATCAACTC |
| P2514_13050up_gib_ura3_R | CTGCCATCATGGTTCAACCTCACCGAGAGCGCACAGAAGTTTCTACAC |
| P2515_pJet_gib_13050down_F | GTTGTGACGCATGCCACATCATCTTTCTAGAAGATCTCCTACAATATTC |
| P2516_pJet_gib_13050up_R | CGGGTGGTCGATCTTGCTGAAAAACTCGAGCCATCCGGAAGATCTG |
| P2517_13050down_gib_ura3_F | GACGATGTGTATAGTGACGATCTGCTGCTAGGAGCTTTGAGCTAAAC |
| P2518_13050down_gib_pJet_R | GGAGATCTTCTAGAAAGATGATGTGGCATGCGTCACAACTAACC |
| P2519_13050up_gib_pJet_F | TGGCTCGAGTTTTTCAGCAAGATCGACCACCCGGAGAACAATCAAG |
| P2537_g13050_KO_cassette_R | TGTGGCATGCGTCACAACTAACC |
| P2538_g13050_KO_cassette_F | CGACCACCCGGAGAACAATCAAG |
| P2550_3'region_13050 _R | GGAAGGGAGCGAGTTCAGAG |
| P2551_5'region_13050_F | GAGAATGGCCACGAAACAAC |
| TP28_01060down_gib_pJet_R | GGAGATCTTCTAGAAAGATTAGGCTTCCACGTCTACTC |
| TP29_01060down_gib_ura3_F | CGATGTGTATAGTGACGATCTTAGAAGAGTCTCCATAAATTAAC |
| TP30_pJet_gib_01060down_F | GGGCTGGAGTAGACGTGGAAGCCTAATCTTTCTAGAAGATCTCCTACAATATTC |
| TP31_pJet_gib_01060up_R | GTGAGTTTGAAGCCCATTCGCTATCTTGCTGAAAAACTCGAGCCATC |
| TP32_01060up_gib_pJet_F | CCGGATGGCTCGAGTTTTTCAGCAAGATAGCGAATGGGCTTCAAACTCAC |
| TP33_01060up_gib_ura3_R | GCCATCATGGTTCAACCTCACCTCAAGTTCGAAAACGGCCAATGTAAC |
| TP34_ura3_gib_01060down_R | GGAGACTCTTCTAAGATCGTCACTATACACATCGTCATCAACTC |
| TP35_ura3_gib_01060up_F | GCCGTTTTCGAACTTGAGGTGAGGTTGAACCATGATGGCAGTGTTC |
| TP36_01060_KO_cassette_R | TAGGCTTCCACGTCTACTC |
| TP37_01060_KO_cassette_F | AGCGAATGGGCTTCAAACTC |
| TP61_3'region_01060_R | CAGCGCTTACAGGGATACAC |
| TP62_5'region_01060_F | CTATGACCCAGATGCTAAGG |
| TP1178_01g03280up_hyg_fw | ccttgcgttaggtacgATGAACAAACGACCCAACACC |
| TP1179_hyg_01g03290down_rev | taaggaacggaggcaCCAATGGCAGTGGCTTACCACTC |
| TP1180_hyg_01g03290down_fw | TAAGCCACTGCCATTGGtgcctccgttccttatctc |
| TP1181_01g03290down_Vbb_rev | GGAGATCTTCTAGAAAGATactagttagtgctcaattttg |
| TP1182_01g03290down_Vbb_fw | aattgagcactaactagtATCTTTCTAGAAGATCTCCTACAATATTC |
| TP1183_Vbb_01g03280_rev | acatcgccgaaagtATCTTGCTGAAAAACTCGAGCCATCC |
| TP1184_Vbb_01g03290_fw | GTTTTTCAGCAAGATactttcggcgatgtgtctg |
| TP1185_01g03280up_hyg_rev | TGGGTCGTTTGTTCATcgtacctaacgcaaggttgac |
| TP1186_01g03280KOlincass_fw | actttcggcgatgtgtctg |
| TP1187_01g03280KOlincass_rev | ACTAGTTAGTGCTCAATTTTG |
| TP1219_01g03280KOup_fw | GAGTGGAAGAAGGAGTATGG |
| TP1220_01g03280KOdown_rv | CAGTGGCAAGGCCAAATATG |

**Table S5** Maximum specific growth rate (µ) of selected *S. bombicola* ABC transporter knockouts cultivated on SD medium.

| **Strain** | **Max specific growth rate (µ)** | **p-value*** |
| --- | --- | --- |
| *S. bombicola* wild type | 0.096 ±0.001 |  |
| *S. bombicola ΔSbCdr4* | 0.052±0.002 | 0.001 |
| *S. bombicola ΔSbYor1.1* | 0.056±0.001 | 0.001 |
| *S. bombicola ΔSbYcf1.2* | 0.067±0.004 | 0.001 |
| *S. bombicola ΔSbYor1.2* | 0.050±0.002 | 0.001 |

* As determined by one-way analysis of variance (ANOVA) with Tukey’s multiple comparisons test. The difference is considered significant when p<0.05.

**Table S6**. List of compounds used for disc diffusion assay, their concentration in DMSO and supplier.

| **Compound** | **Concentration** | **Supplier** |
| --- | --- | --- |
| **Flavonoids** |  |  |
| Myricetin | 135 mg/mL | Carbosynth Limited |
| Quercetin | 300 mg/mL | Sigma-Aldrich |
| Lutiolin | 135 mg/ mL | Carbosynth Limited |
| Daidzein | 65 mg/mL | Carbosynth Limited |
| Phloretin | 135 mg/mL | Carbosynth Limited |
|  |  |  |
| **Miscellaneous compounds** |  |  |
| Rhodamine B | 10 mg/mL | Sigma-Aldrich |
| 2-nitrophenol | 10% (v/v) | Sigma-Aldrich |
| Aniline | 1900 mg/mL | Sigma-Aldrich |
|  |  |  |
| **Detergents** |  |  |
| Polyoxyethylene 9 Lauryl ether | 2% (v/v) | Sigma-Aldrich |
| Triton X-100 | 12.5% (v/v) | Sigma-Aldrich |
| Triton X-144 | 12.5% (v/v) | Sigma-Aldrich |
| Lauroylsarosine, sodium salt | 10 mg/mL | Sigma-Aldrich |
|  |  |  |
| **Long chain fatty acids and hydroxylated fatty acids** | | |
| Arachidic Acid | 1.7 mg/mL | Sigma-Aldrich |
| Behenic Adic | 1.7 mg/mL | Sigma-Aldrich |
| 16-hydroxyhexadecanoic acid | 2% (v/v) | Sigma-Aldrich |
| 3-hydroxydecanoic acid | 2% (v/v) | Sigma-Aldrich |
|  |  |  |
| **Long, medium and “special” chain alkanes** | | |
| 1,2-Epoxyotane | 2% (v/v) | Sigma-Aldrich |
| Dotriacontane | 25 mg/mL | Acros Organics |
| Pristane | 2% (v/v) | Sigma-Aldrich |
| (Poly)propylene carbonate | 5% (v/v) | Sigma-Aldrich |
| Decane | 1% (v/v) | Sigma-Aldrich |
| undecane | 1% (v/v) | Sigma-Aldrich |
| dodecane | 1% (v/v) | Sigma-Aldrich |
|  |  |  |
| **Fatty alcohols** |  |  |
| 1-Hexanol | 1% (v/v) | Sigma-Aldrich |
| 1-octanol | 1% (v/v) | Sigma-Aldrich |
| 1-Decanol | 1% (v/v) | Sigma-Aldrich |
| Undecanol | 1% (v/v) | Sigma-Aldrich |
| Dodecanol | 1% (v/v) | Sigma-Aldrich |
| 1-tetradecanol | 2% (v/v) | Sigma-Aldrich |
| 1-Hexadecanol | 0.83 mg/mL | Sigma-Aldrich |
| 2-Hexadecanol | 0.83 mg/mL | Sigma-Aldrich |
| 1-Octadecanol | 0.83 mg/mL | Sigma-Aldrich |
| Oleyl alcohol | 0.83 mg/mL | Sigma-Aldrich |
| 2-Decyl-1-tetradecanol | 2% (v/v) | Sigma-Aldrich |
| **Antibiotics (in liquid culture)** |  |  |
| G418 | 600 µg/mL | Thermo-Fischer |
| Hygromycin B | 400 µg/mL | Sigma-Aldrich |
| Chloramphenicol | 300 µg/mL | Sigma-Aldrich |
| Nystatin | 80 µg/mL | Sigma-Aldrich |
| Phleomycin | 200 µg/mL | Sigma-Aldrich |


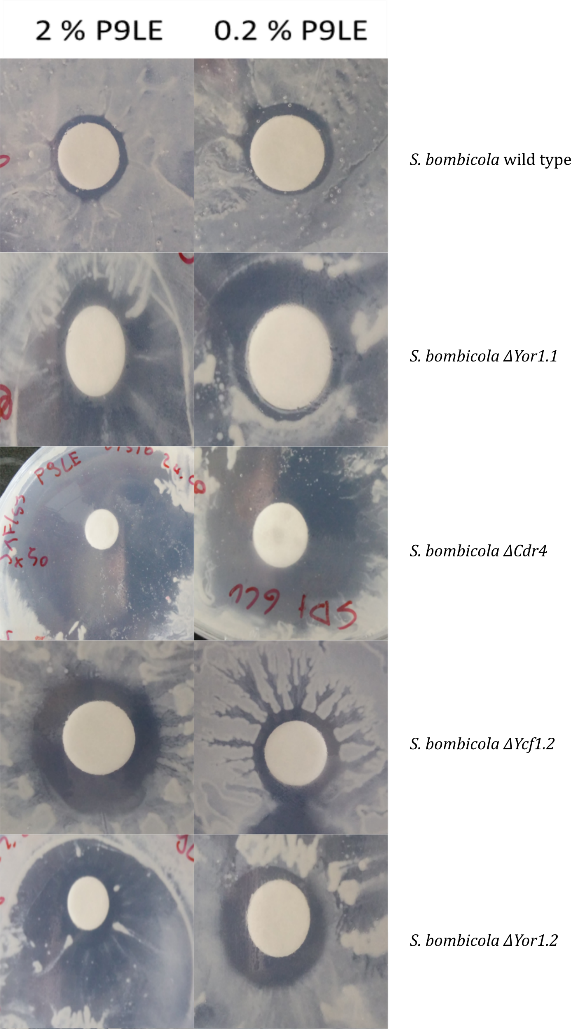


**Figure S1** Disc diffusion assay with polyoxyethylene-9-lauryl ether of selected *S. bombicola* ABC transporter deletion mutants compared to wild type.


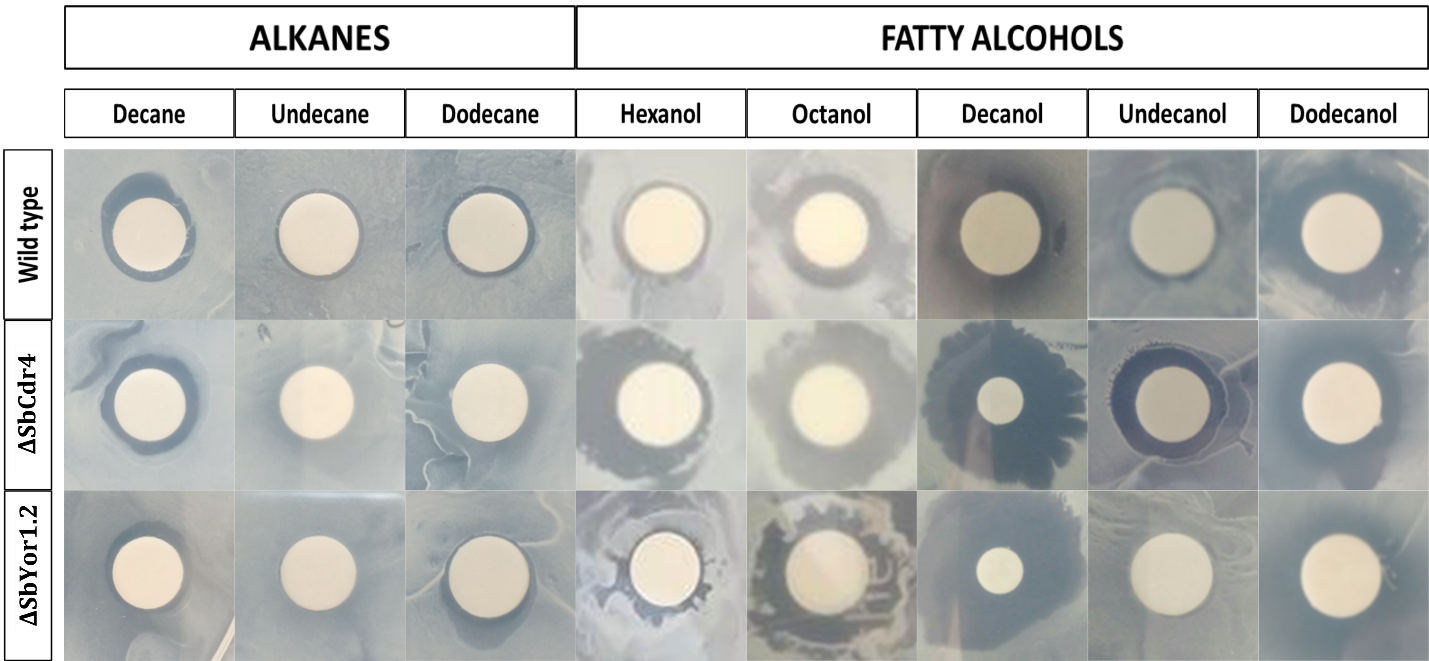


**Figure S2** Disc diffusion assay of *S. bombicola ΔSbCdr4* and *S. bombicola ΔSbYor1.2* compared to wild type. The concentration of the applied medium chain alkanes and fatty alcohols is given in table S6.


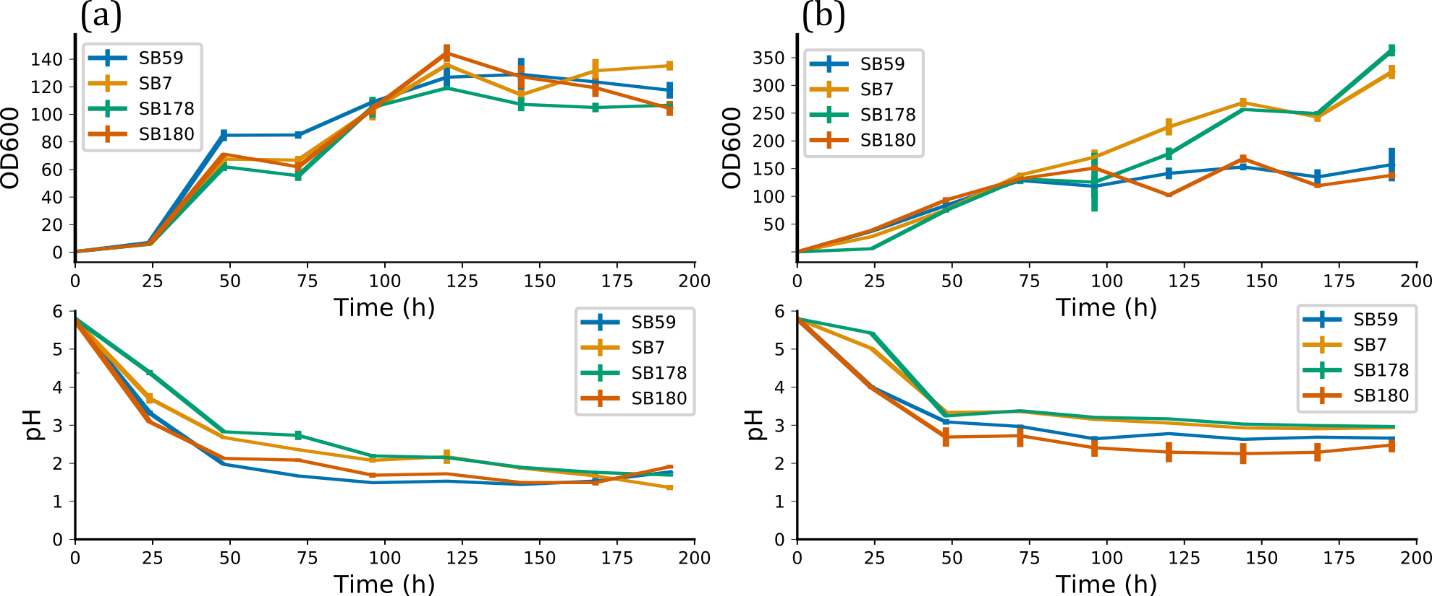


**Figure S3** Comparison of growth among different SL transporter deletion strains on shake flask scale by OD_600_ and pH measurements every 24h (a) without the addition of rapeseed oil and (b) with the addition of 37.5 g/L rapeseed oil.

**References**

1. Mishra NK, Chang J, & Zhao PX (2014). Prediction of membrane transport proteins and their substrate specificities using primary sequence information. *PLoS One*. 9, 3-6.

2. Van Bogaert I, De Maeseneire S, Develter D, Soetaert W, & Vandamme EJ (2008). Cloning and characterisation of the glyceraldehyde 3-phosphate dehydrogenase gene of *Candida bombicola* and use of its promoter. *J Ind Microbiol Biotechnol*. 35, 1085-1092.

3. Van Bogaert INA, Holvoet K, Roelants SLKW, et al. (2013). The biosynthetic gene cluster for sophorolipids: a biotechnological interesting biosurfactant produced by *Starmerella bombicola*. *Mol Microbiol*. 88, 501-509.
